# Rab6 Is Required for Multiple Apical Transport Pathways but Not the Basolateral Transport Pathway in *Drosophila* Photoreceptors

**DOI:** 10.1371/journal.pgen.1005828

**Published:** 2016-02-18

**Authors:** Nozomi Iwanami, Yuri Nakamura, Takunori Satoh, Ziguang Liu, Akiko K. Satoh

**Affiliations:** 1 Division of Life Science, Graduate School of Integral Arts and Science, Hiroshima University, Higashi-Hiroshima, Japan; 2 Institute of Animal Husbandry, Heilongjiang Academy of Agricultural Sciences, Harbin, Heilongjiang, China; University of Cambridge, UNITED KINGDOM

## Abstract

Polarized membrane trafficking is essential for the construction and maintenance of multiple plasma membrane domains of cells. Highly polarized *Drosophila* photoreceptors are an excellent model for studying polarized transport. A single cross-section of *Drosophila* retina contains many photoreceptors with 3 clearly differentiated plasma membrane domains: a rhabdomere, stalk, and basolateral membrane. Genome-wide high-throughput ethyl methanesulfonate screening followed by precise immunohistochemical analysis identified a mutant with a rare phenotype characterized by a loss of 2 apical transport pathways with normal basolateral transport. Rapid gene identification using whole-genome resequencing and single nucleotide polymorphism mapping identified a nonsense mutation of Rab6 responsible for the apical-specific transport deficiency. Detailed analysis of the trafficking of a major rhabdomere protein Rh1 using blue light-induced chromophore supply identified Rab6 as essential for Rh1 to exit the Golgi units. Rab6 is mostly distributed from the *trans*-Golgi network to a Golgi-associated Rab11-positive compartment that likely recycles endosomes or transport vesicles going to recycling endosomes. Furthermore, the Rab6 effector, Rich, is required for Rab6 recruitment in the *trans*-Golgi network. Moreover, a Rich null mutation phenocopies the Rab6 null mutant, indicating that Rich functions as a guanine nucleotide exchange factor for Rab6. The results collectively indicate that Rab6 and Rich are essential for the *trans*-Golgi network–recycling endosome transport of cargoes destined for 2 apical domains. However, basolateral cargos are sorted and exported from the *trans*-Golgi network in a Rab6-independent manner.

## Author Summary

Cells in animal bodies have multiple plasma membrane domains; this polarized characteristic of cells is essential for their specific functions. Selective membrane transport pathways play key roles in the construction and maintenance of polarized structures. *Drosophila* photoreceptors with multiple plasma membrane domains are an excellent model of polarized transport. We performed genetic screening and identified a Rab6 null mutant with a rare phenotype characterized by a loss of 2 apical transport pathways with normal basolateral transport. Although Rab6 functions in the Golgi are well known, its function in polarized transport was unexpected. Here, we found that Rab6 and its effector, Rich, are required for multiple apical transport pathways but not the basolateral transport pathway. Our findings strongly indicate that the membrane proteins delivered to multiple polarized domains are not sorted simultaneously: basolateral cargos are segregated before the Rab6-dependent process, and cargos going to multiple apical domains are sorted after Rab6-dependent transport from the *trans*-Golgi network to the Golgi-associated Rab11-positive compartment, which presumably recycles endosomes. Our finding of the function of Rab6 in polarized transport will elucidate the molecular mechanisms of polarized transport.

## Introduction

**Fig 1:**
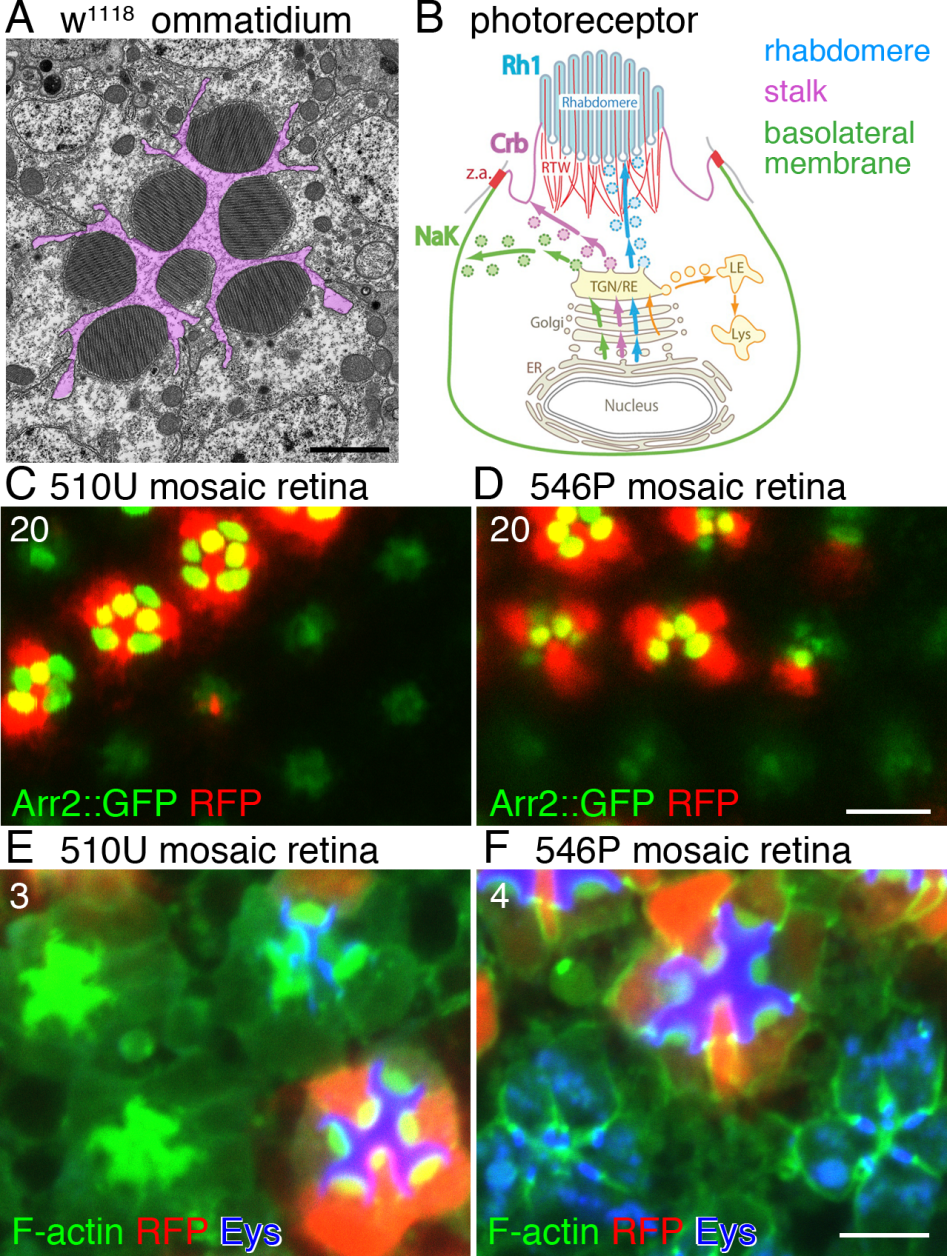
Screening and identification of mutations with a closed rhabdomere phenotype. (A) Cross-section of an ommatidium from a w^1118^ late pupal stage observed by electron microscopy. Purple shading shows the inter-rhabdomeric space. (B) Schematic view of a cross section of a photoreceptor showing 3 different plasma membrane domains: the rhabdomere, stalk, and basolateral membrane. (C, D) 510U (C) and 546P (D) mosaic retinas. RFP (red) indicates wild type cells, and Arrestin2::GFP (green) shows endogenous Rh1 localization. (E, F) Immunostaining of ommatidial cross-sections of 510U (E) and 546P (F) mosaic retinas. F-actin and anti-Eys antibody stainings are shown in green and blue, respectively. RFP (red) indicates wild type cells. Scale bars: 2 μm (A), 10 μm (C, D), 5 μm (E, F) 5 μm. Numbers of the samples observed were shown in the top-left corner of the images.

Many cells that make up animal bodies have multiple plasma membrane domains, which are the basis of their specific functions. Polarized vesicle transport is essential for establishing and maintaining these domains. However, the underlying mechanisms are not well elucidated. The *Drosophila* retina is a good genetics-based model system for studying polarized transport. A single retina contains approximately 800 ommatidia, which comprise 6 outer and 2 central photoreceptors with 4 distinct plasma membrane domains (Fig 1A and 1B, see also [1]). The first domain is the photoreceptive membrane domain, i.e., the rhabdomere, which is formed at the center of the apical plasma membrane as a column of closely packed rhodopsin-rich photosensitive microvilli. The second is the peripheral apical domain surrounding the rhabdomere, i.e., the stalk membrane, which is enriched in Crb and beta H-spectrin [2, 3]. “Eye shut” (Eys) is an extracellular matrix protein secreted from the stalk domain of photoreceptors from the early to mid-pupal stages [4, 5]; its secretion is necessary and sufficient to form the inter-rhabdomeric space (IRS). The third domain is the basolateral membrane, which is separated from the apical membrane by adherens junctions, where Na^+^K^+^ATPase localizes, similar to typical polarized epithelial cells [6]. Finally, the fourth domain comprises the axon and synapses, which extend below the retina to the brain.

Rh1, the rhodopsin expressed in R1–6 outer retinal photoreceptor cells, is the most accessible protein for investigating apical polarized transport in *Drosophila* photoreceptors. Blue light-induced chromophore supply (BLICS) releases Rh1 from the endoplasmic reticulum (ER) [7], allowing us to trace synchronous Rh1 trafficking. We previously used this method to show that Rab1 is involved in ER–Golgi transport [8], that GPI synthesis is essential for Rh1 sorting in the *trans*-Golgi network (TGN) [1], and that the Rab11/dRip11/MyoV complex is essential for post-Golgi Rh1 transport [9, 10]. In addition, other studies demonstrate that the exocyst complex tethers post-Golgi vesicles to the base of the rhabdomeres [11] and that Rab6Q71L overexpression inhibits Rh1 transport in the early secretory pathway [12].

Rab6 is a member of the Rab family of small GTPases, which regulate the specificity between donor and acceptor membranes for vesicle budding, docking, tethering, and fusion steps during transport [13–15]. Human Rab6 comprises 4 different isoforms—Rab6a, Rab6a′, Rab6b, and Rab6c—and is the most abundant Golgi-associated Rab protein [16]. Rab6 regulates retrograde transport from the Golgi complex to the ER [17, 18]; the retrograde transport of the B subunit of Shiga toxin from early endosomes or recycling endosomes (REs) to the Golgi complex [19, 20]; and the post-Golgi trafficking of herpes simplex virus 1 (HSV1) envelop proteins, tumor necrosis factor (TNF), and vesicular stomatitis virus G-protein (VSV-G) to the plasma membrane [21–23].

In the present study, we screened ethyl methanesulfonate (EMS)-mutagenized flies to find mutants with defective polarized transport. As one of the mutants identified was a null allele of Rab6, we reevaluated the function of Rab6 in *Drosophila* photoreceptors and found Rab6 is essential for transport towards both distinct apical domains (i.e., the stalk and rhabdomeres) but not towards the basolateral membrane. These results indicate basolateral cargos are sorted and exported from the TGN in a Rab6-independent manner before they are sorted into pathways destined for the stalk and rhabdomeres.

## Results

### Live-image screening of EMS-mutagenized mutants exhibiting rhabdomere morphogenesis in *Drosophila* photoreceptors

To identify the genes essential for rhabdomere morphogenesis and membrane trafficking, we previously performed retinal mosaic screening of P-element–inserted lines maintained in stock centers [24] using the FLP/FRT method [25] with *in vivo* fluorescent imaging [26] of Arrestin2::GFP [1]. To extend the screening to comprehensive coverage, we are currently engaged in genome-wide, large-scale screening of EMS-mutagenized chromosomes; thus far, we have isolated 233 Rh1-accumulation deficient mutant lines, which stochastically covers more than 80% of genes in more than 60% of *Drosophila* genome [27].

Flies including *Drosophila melanogaster* have an open rhabdom system, in which the rhabdomeres of each ommatidium are separated from each other and function as independent light guides. In contrast, bees and mosquitoes have a closed system, in which rhabdomeres within each ommatidium are fused to each other, thus sharing the same visual axis. Without expression or secretion to the IRS of the extracellular matrix glycoprotein Eys, fly ommatidia exhibit a closed rhabdom [4, 5]. By screening EMS-mutagenized left arms of the second chromosome, we isolated 11 mutant lines with a closed rhabdom phenotype similar to the *eys* mutant (Fig 1C and 1D); these lines were further classified into 4 complementation groups. The mutations in one group failed to complement a loss-of-function allele of the *eys* gene, *eys*^*BG02208*^ [4], indicating that mutations in this group represent new loss-of-function alleles of *eys*. On the other hand, the mutations in the other 3 groups complemented the *eys*^*BG02208*^ mutant. Fig 1C and 1D show Arrestin2-GFP localizations in 510U mutant in the *eys* complementation group as well as 546P in another complementation group; 546P was the sole member of the complementation group, and the homozygotes were lethal in the early larval stage. The closed rhabdom phenotypes of 510U and 546P had 100% penetrance: all adult flies observed for 510U or 546P using Arr2GFP had mutant ommatidia with a fused rhabdomere. We subsequently observed similar high penetrance of the phenotypes in immunostaining at the fly and cell levels unless otherwise noted.

Immunostaining of the mosaic retina of these mutants with anti-Eys antibody revealed complete loss of Eys protein in the whole mutant ommatidia of 510U (Fig 1E), which again strongly suggests the complementation group (which contains 510U) represents *eys*. On the other hand, in 546P mutant ommatidia, Eys was readily detected; however, it did not accumulate in the extracellular domain (i.e., the IRS) but rather within undefined cytoplasmic structures (Fig 1F). These results suggest the transport of Eys protein is inhibited in the 546P mutant.

**Fig 2:**
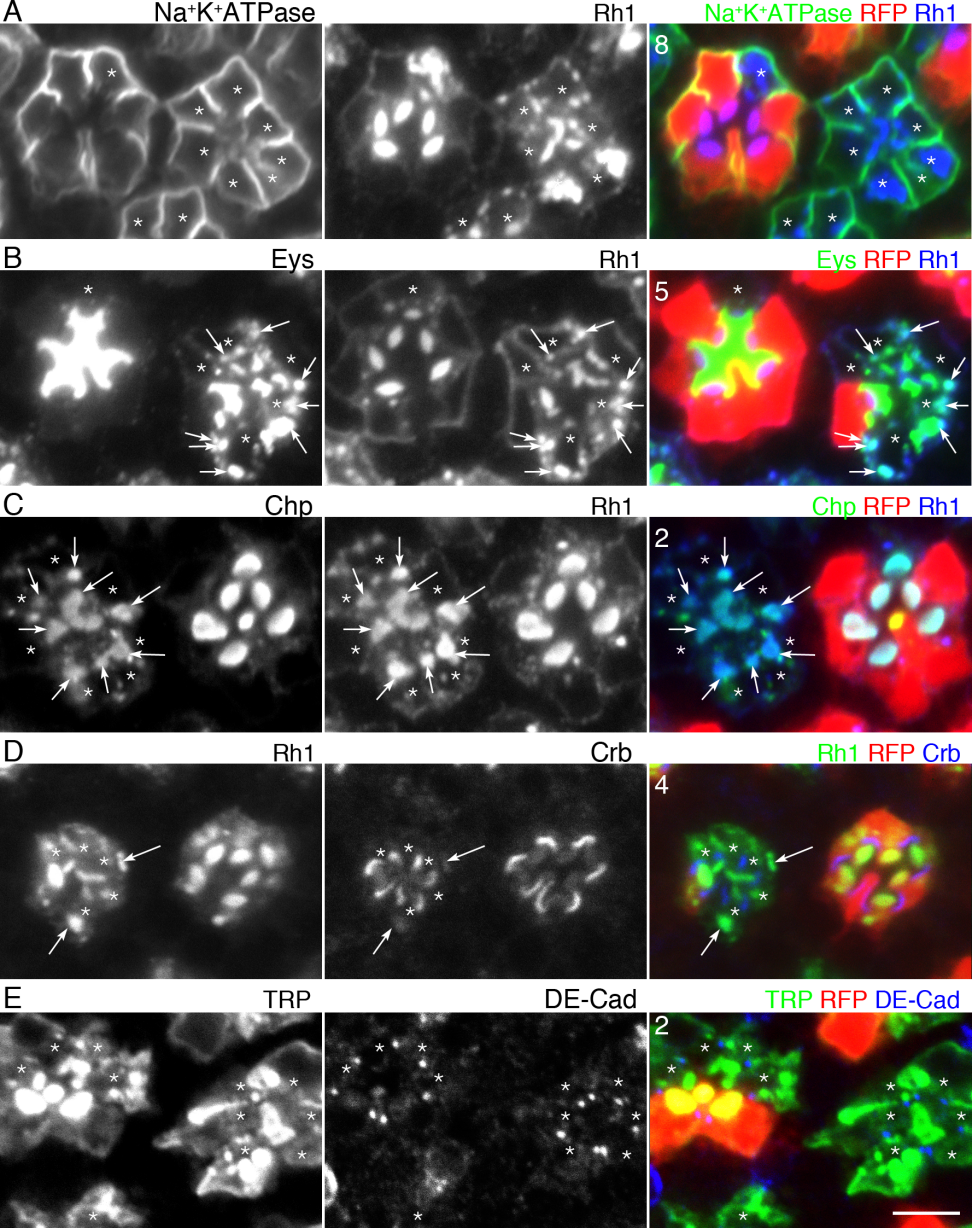
Membrane protein localizations in 546P mutant mosaic eyes. 546P mutant mosaic eyes immunostained by the following antibodies and phalloidin. RFP (red) indicates wild type cells. Asterisks show 546P mutant photoreceptors. (A) Anti-Rh1 (blue) and anti-Na^+^K^+^ATPase antibodies (green). (B) Anti-Rh1 (blue) and anti-Eys antibodies (green). Rh1 co-localized with Eys in the cytoplasm (white arrows). (C) Anti-Rh1 (blue) and anti-Chp antibodies (green). Chp co-localized with Rh1 in the cytoplasm (white arrows). (D) Anti-Crb antibody (blue) and anti-Rh1 (green). Crb co-localized with Rh1 in the cytoplasm (white arrows). (E) Anti–DE-Cad (blue) and anti-TRP antibodies (green). Scale bars: 5 μm (A–E). Numbers of the samples observed were shown in the top-left corner of the composite images.

We subsequently investigated the localization of several plasma membrane proteins in 546P mutant photoreceptors (Fig 2). In wild type photoreceptor cells, Rh1 localized at the photoreceptive rhabdomeres. However, Rh1 was mainly detected in the undefined cytoplasmic structures in 546P mutant photoreceptors (Fig 2A, blue). In contrast to Eys and Rh1, a basolateral membrane protein, Na^+^K^+^ATPase localized normally in the basolateral membrane and did not accumulate in the cytoplasm of 546P mutant photoreceptors (Fig 2A, green). Eys and Rh1 were co-localized in the same undefined cytoplasmic structures (Fig 2B). Two other rhabdomeric proteins—TRP and Chaoptin (Chp)—and a stalk protein—crumbs (Crb)—were also detected in the undefined cytoplasmic structures (Fig 2C–2E). On the other hand, DE-Cadherin (DE-Cad) was localized normally at the adherens junctions (Fig 2E). These results indicate inhibition of transport toward the rhabdomeres and stalks but not toward the basolateral membrane. Coinciding with the localizations of plasma membrane proteins, the basolateral membrane in 546P mutant photoreceptors was extended as in the wild type despite the shrunken stalk membrane and small rhabdomeres (Figs 2A, 2D and 2E and S5). None of the 222 other Rh1 accumulation-deficient lines clearly showed 546P-like phenotypes, closed rhabdomeres, or inhibition of transport toward the rhabdomere and stalk (but not toward the basolateral membrane), indicating these 546P phenotypes are characterized by an apical-specific transport deficiency rather than the result of a delay or loss of general membrane transport.

**Fig 3:**
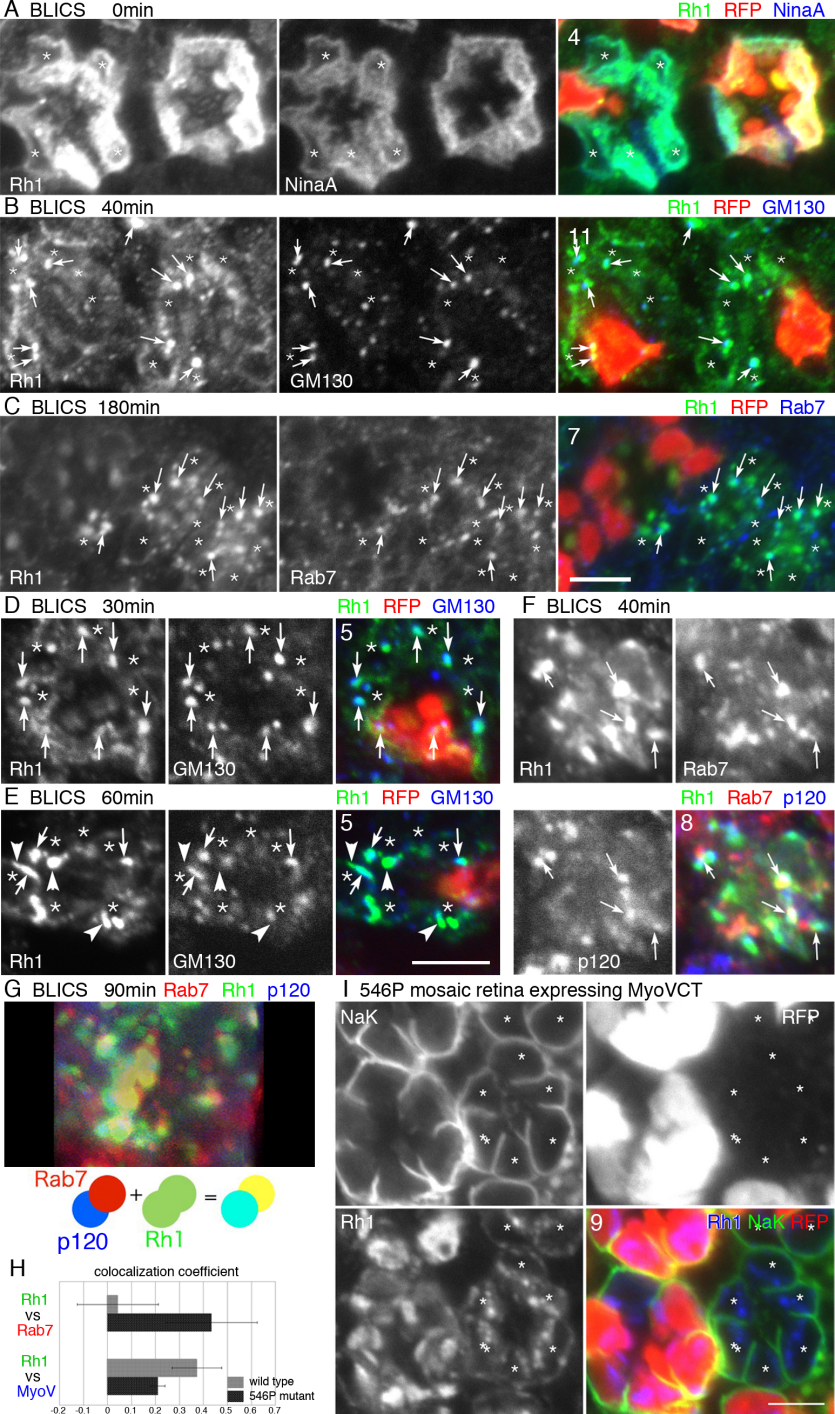
Kinetics of Rh1 transport in 546P mutant photoreceptors. 546P mutant mosaic eye immunostained by the following antibodies. RFP (red) indicates wild type cells. Asterisks show 546P mutant photoreceptors. (A) Immunostaining before BLICS. Anti-Rh1 (green) and anti-NinaA antibodies (blue, endoplasmic reticulum marker). (B) Immunostaining 40 min after BLICS. Anti-Rh1 (green) and anti-GM130 (blue, Golgi marker) antibodies. (C) Immunostaining 180 min after BLICS. Anti-Rh1 (green) and anti-Rab7 (blue, late endosome marker) antibodies. (D, E) Immunostaining 30 min (D) and 60 min (E) after BLICS. Anti-Rh1 (green) and anti-GM130 (blue, Golgi marker) antibodies. (F) Immunostaining of a 546P mutant ommatidium 90 min after BLICS. Anti-Rh1 (green), Rab7 (red, late endosome marker) and anti-p120 (blue, Golgi marker) antibodies. (G) Projection image from 20 slices at 0.5-μm intervals of 546P mutant ommatidium 90 min after BLICS. Anti-Rh1 (green), Rab7 (red) and anti-p120 (blue) antibodies. A schematic view of Rh1, Rab7, and p120 is shown under the projection image. (H) Pearson’s correlation coefficients of the co-localization between Rh1 and Rab7, and Rh1 and MyoV as a control. MyoV predominantly localizes on the pigment granules in the photoreceptors. (I) Immunostaining of 546P mutant mosaic eye expressing GFP::MyoV C-terminal dominant-negative protein by Rh1Gal4 driver. Asterisks show 546P mutant photoreceptors. Scale bars: 5 μm (A–G, I). Numbers of the samples observed were shown in the top-left corner of the composite images.

To clarify the details of the transport defects in 546P mutant photoreceptors, we investigated the dynamics of Rh1 transport using BLICS [1, 8]. Rh1 comprises an apoprotein, opsin, and the chromophore *11-cis* retinal. Without the chromophore, opsin accumulates in the ER. Blue light illumination photoisomerizes *all-trans* retinal to *11-cis*, inducing the synchronous release of Rh1 from the ER into the secretory pathway. Prior to BLICS, Rh1-apoprotein accumulated normally in the ER and Rh1 was transported to the Golgi units by 40 min after BLICS in 546P mutant photoreceptors (Fig 3A and 3B). However, in contrast to wild type photoreceptors, Rh1 did not reach the rhabdomeres in 546P mutant photoreceptors but instead localized in large globular structures in the cytoplasm with the late endosome marker Rab7 180 min after BLICS (Fig 3C and 3H). These results indicate Rh1 is normally synthesized in the ER and transported to the Golgi units in 546P mutant photoreceptors but is subsequently transported to the late endosomes rather than the rhabdomeres.

To determine how Rh1 is exported from the Golgi units to the late endosomes, we compared Rh1 staining in the Golgi units 30 and 60 min after BLICS in both wild type and 546P mutant photoreceptors (Fig 3D and 3E). There were no apparent differences between wild type and 546P mutant photoreceptors 30 min after BLICS. However, 60 min after BLICS, Rh1 staining became stronger in the Golgi units of mutant photoreceptors than wild type photoreceptors. Moreover, the Golgi units extended Rh1-positive tubular or globular structures (Fig 3E). Next, we triple stained 546P mutant photoreceptors with antibodies against the medial-Golgi marker p120 [28], late endosomal marker Rab7 [29], and Rh1 40 and 90 min after BLICS and investigated the Rh1-positive tubular or globular structures extending from the Golgi units (Figs 3F and S1). Golgi markers p120, Rab7, and Rh1 were frequently partially co-localized. Rab7 staining was often adjacent to p120 staining, and Rh1 staining overlapped with both of them (Fig 3F, arrows; S1 Fig). A Z-projection (Fig 3G) of 20 optical sections at 0.5-μm intervals (S1 Fig) showed most Golgi units faced the globular Rab7-positive structure, both of which were positive for Rh1; the Golgi units with Rh1 (turquoise) were often adjacent/connected to late endosomes containing Rh1 (yellow). These observations suggest Rh1 is first transported to the Golgi units in a normal manner but is not exported normally and subsequently accumulates there; finally, Rh1 directly exits the Golgi units to Rab7-positive tubular or globular structures.

### The 546P mutation is epistatic to a MyoV mutation

We previously showed that the Rab11/dRip11/MyoV complex is essential for post-Golgi vesicle transport and that deficiency of any component of the complex induces cytoplasmic accumulation of Rh1-loaded post-Golgi vesicles [9, 10]. The pattern of Rh1-accumulation observed in the 546P mutant clearly differed from those of Rab11, dRip11, or MyoV mutants.

To investigate the epistatic interaction between mutations of the Rab11/dRip11/MyoV complex and the 546P mutation for Rh1 transport, we observed Rh1 localization in 546P mutant mosaic retina expressing the dominant-negative MyoV C-terminal domain. Rh1 did not accumulate in the cytoplasm of 546P/MyoV double-mutant photoreceptors like the MyoV single mutant but instead accumulated in the globular structures (Fig 3H). This phenotype was indistinguishable from that of cells with only the 546P mutation (Fig 2A–2C). This result indicates the 546P mutation is epistatic to mutations of the Rab11/dRip11/MyoV complex. Thus, in the 546P mutant, the Rh1 in Rab7-positive late endosomes directly comes from the Golgi units before being sorted into post-Golgi vesicles.

Kinetic and epistatic analyses of Rh1 transport in 546P mutant cells revealed that the processes between Golgi entry and before/upon post-Golgi vesicle formation are inhibited in the 546P mutant during Rh1 biosynthetic trafficking.

### A nonsense mutation of Rab6 is responsible for the 546P phenotype

As the phenotype of 546P is drastic and important for understanding the mechanism of polarized vesicle transport, we identified the mutation responsible for the phenotype. Rough mapping using meiotic recombination and restriction fragment length polymorphisms (RFLP) [30] placed the 546P mutation between RFLP893 and RFLP977. We subsequently sequenced the whole genome of 546P homozygous animals using whole-genome amplification and a next-generation sequencer [27] and found 4 unique mutations in the mapped area; complementation tests over defined chromosomal deletions showed that only one of these, a nonsense mutation on the Rab6 gene (Rab6 R73 to a stop codon), is lethal like 546P homozygous larvae.

**Fig 4:**
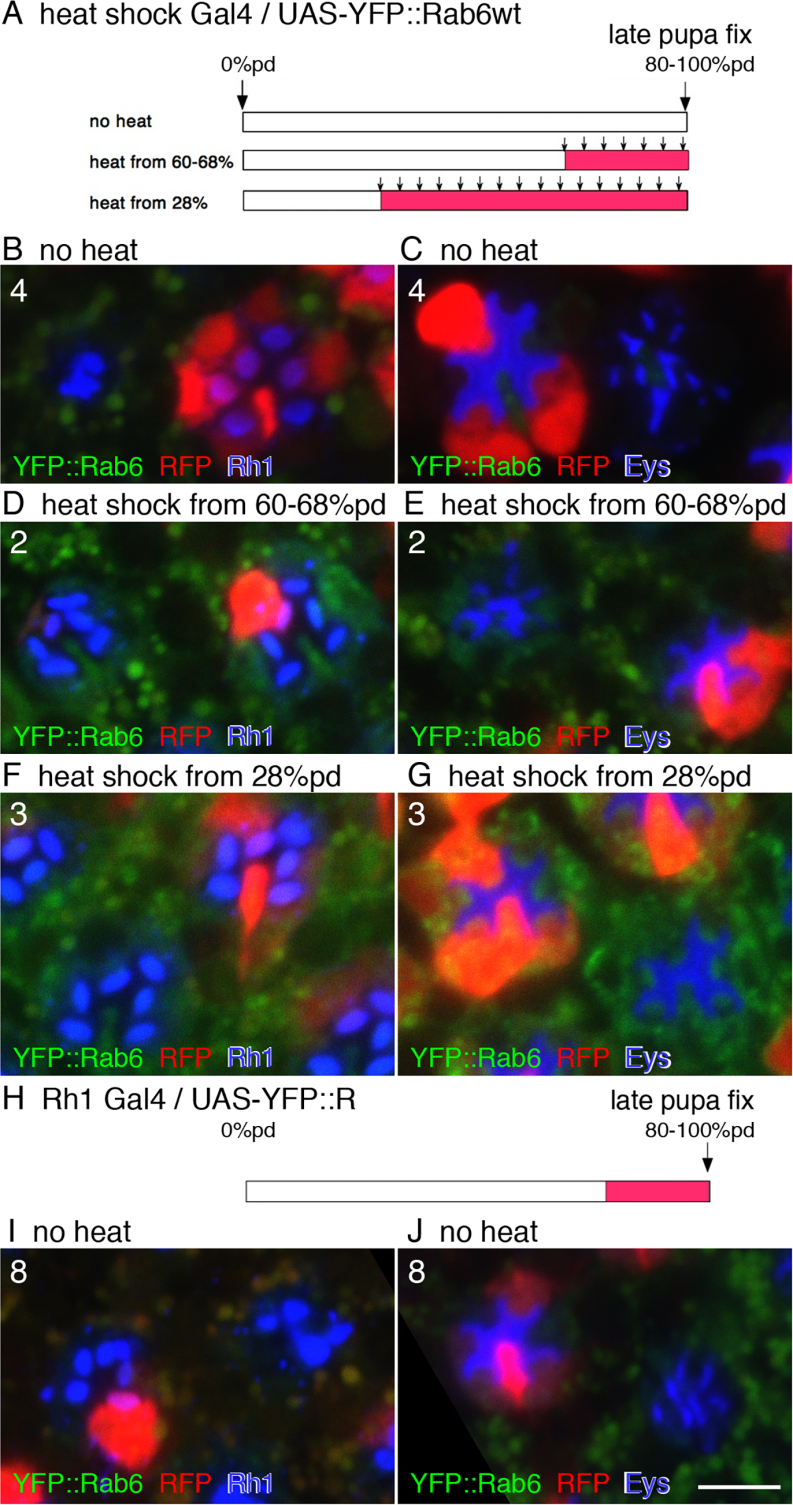
YFP::Rab6wt expression rescues the 546P mutant phenotype. (A–G) 546P mutant mosaic eyes expressing YFP::Rab6wt driven by heat shock-Gal4 were immunostained with the indicated antibodies. RFP (red) indicates wild type cells. (A) Schematic of the heat shock procedure (arrow) and YFP::Rab6wt expression (pink bar). (B, D, F) Immunostaining of 546P mutant mosaic eyes expressing YFP::Rab6wt driven by heat shock-Gal4. YFP::Rab6wt and Rh1 are shown in green and blue, respectively. Significant autofluorescence was also observed in pigment granules in the green channel. (C, E, G) Immunostaining of a 546P mutant mosaic eye expressing YFP::Rab6wt driven by Rh1-Gal4. YFP::Rab6wt with auto-fluorescence of pigment granules and Eys are shown in green and blue respectively. Significant autofluorescence was also observed in pigment granules in the green channel. (H–J) 546P mutant mosaic eyes expressing YFP::Rab6wt driven by Rh1-Gal4 were immunostained with the indicated antibodies. RFP (red) indicates wild type cells. (H) Schematic view of YFP::Rab6wt expression (pink bar). (I) Immunostaining of 546P mutant mosaic eyes expressing YFP::Rab6wt driven by Rh1-Gal4. YFP::Rab6wt with autofluorescence of pigment granules and Rh1 are shown in green and blue, respectively. (J) Immunostaining of 546P mutant mosaic eyes expressing YFP::Rab6wt driven by Rh1-Gal4. YFP::Rab6wt with autofluorescence of pigment granules and Eys are shown in green and blue respectively. Scale bars: 5 μm (A–J). Numbers of the samples observed were shown in the top-left corner of the composite images.

As a Rab6 null mutation was found in the mapped area, we subsequently determined if YFP::Rab6wt expression rescues the 546P phenotype. Using the UAS/Gal4 system, we expressed YFP::Rab6wt from early/late pupa in whole bodies using heat shock-Gal4 or in late pupal outer photoreceptors using Rh1-Gal4 (Fig 4). YFP::Rab6wt expression from 28% pupal development by heat shock-Gal4 completely rescued the 546P phenotype (Fig 4F and 4G), confirming that the Rab6 gene is responsible for the 546P phenotype. Therefore, we designated 546P as a new allele of the Rab6 gene, Rab6^546P^. Interestingly, YFP::Rab6wt expression starting from 68% pupal development by heat shock-Gal4 or by Rh1-Gal4 showed partial rescue; Rh1 accumulated in fused rhabdomeres lacking normal level of Eys in the IRS (Fig 4D, 4E, 4I and 4J). These results are concordant with previous reports on Eys/spacemaker [5] showing that Eys expression in early pupae is required to form open rhabdomeres.

### Defects in the polarized trafficking of membrane proteins in Rab6^D23D^ and Rich^1^ photoreceptors

We subsequently investigated whether photoreceptors homozygous for the other Rab6 null allele Rab6^D23D^ have defects in Rh1 and Eys trafficking. Like Rab6^546P^ mutant photoreceptors, Rab6^D23D^ ommatidia exhibited a closed rhabdomere phenotype visualized by phalloidin staining (S2A Fig). Eys accumulated in the globular cytoplasmic structures, although a limited amount localized between photoreceptor apical membranes (S2A Fig). In Rab6^D23D^ photoreceptors, most Rh1 was localized in the cytoplasm, whereas the basolateral membrane protein Na^+^K^+^ATPase localized normally in the basolateral membrane (S2B Fig). These results corroborate the notion that the 546P phenotype is due to Rab6 protein deficiency.

In yeast and mammalian cells, the Ric1/Rgp1 complex functions as a guanine nucleotide exchange factor (GEF) for Rab6 [31, 32]. The *Drosophila* Ric1p homolog Rich is a Rab6 effector in *Drosophila* [33]. Rich contains RIC1 and WD40 domains, both of which bind to fly Rab6. Although neither the GEF activity for Rab6 nor binding to the *Drosophila* rgp1 homolog CG1116 has been confirmed, Rich might function as a GEF together with other interacting proteins [33]. Rab6 and Rich interact genetically and are required for synaptic specificity in fly eyes and olfactory receptor neurons [33]. Therefore, we investigated the localizations of Rh1 and Eys in photoreceptors homozygous for a null allele in Rich, Rich^1^. Similar to Rab6^546P^ and Rab6^D23D^ photoreceptors, Rh1 and Eys accumulation in the rhabdomeres and IRS, respectively, were limited, and both proteins were detected in cytoplasmic structures (S2C and S2D Fig). On the other hand, the Na^+^K^+^ATPase localized normally in the basolateral membrane in Rich^1^ mutant photoreceptors (S2D Fig). The phenotypes were highly penetrant in Rab6^D23D^ photoreceptors. However, in the case of Rich^1^ homozygous photoreceptors, some Rich^1^ mutant cells only exhibited a weak phenotype.

Detailed observation of Rh1 transport by BLICS in Rab6^D23D^ and Rich^1^ mutants showed that Rh1 is synthesized and transported to the Golgi units in a normal manner; however, after leaving the Golgi units, most Rh1 enters the late endosomes instead of the rhabdomeres in the same manner as in Rab6^546P^ photoreceptors (S3 Fig). The localizations of other membrane proteins were investigated in Rab6^D23D^ and Rich^1^ mutants (S4 Fig). The rhabdomeric proteins Chp and TRP accumulated in the cytoplasm in both Rab6^D23D^ and Rich^1^ mutants. The localization of the stalk protein Crb in the stalk membrane was limited in both Rab6^D23D^ and Rich^1^ mutants, but its cytoplasmic accumulation was observed only in Rab6^D23D^ mutants. More severe membrane trafficking defects were observed in Rab6^D23D^ mutants than Rich^1^ mutants. On the other hand, DE-Cad localized normally in the basolateral membrane in both Rab6^D23D^ and Rich^1^ mutants. These observations coincide with the Rab6^546P^ phenotype. Quantification of the lengths of the stalk and basolateral membrane in tangential sections revealed that Rab6^546P^ and Rab6^D23D^ mutant photoreceptors had shorter stalk membranes than those of wild type but basolateral membranes of normal length (S5A–S5F Fig).

Furthermore, we determined if Rab6 deficiency affects the transport of another basolateral protein, FasIII, using ovarian follicle cells (S6 Fig). FasIII localized normally on the basolateral membrane, whereas the localization of the apical membrane protein, Notch, was diminished in follicle cells. Thus, the involvement of Rab6 in apical transport but not basolateral transport could be common in polarized cells.

### Multi-vesicular bodies are increased in Rab6^546P^, Rab6^D23D^, and Rich^1^ mutant photoreceptors

**Fig 5:**
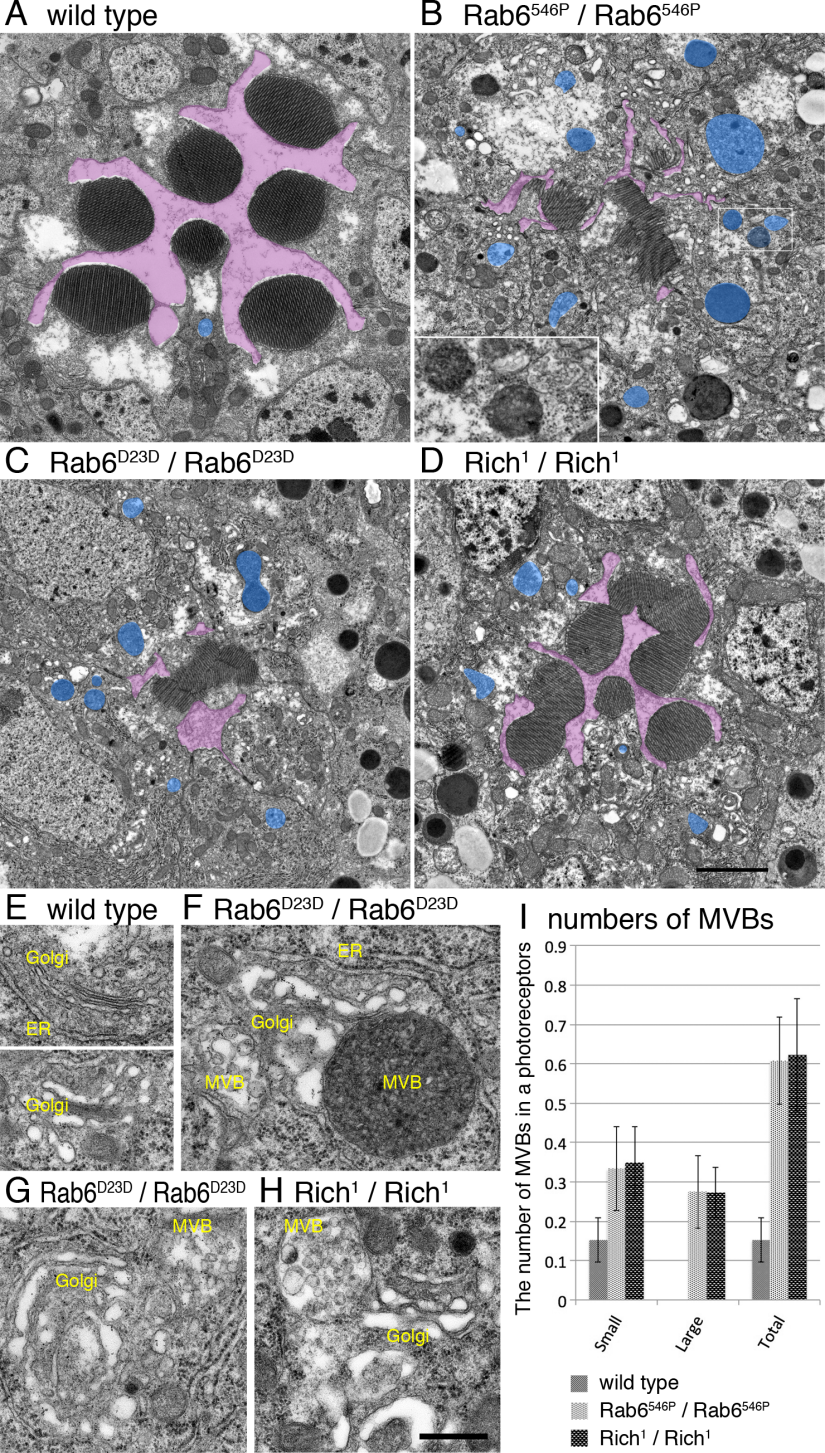
MVBs are increased in Rab6^546P^, Rab6^D23D^, and Rich^1^ mutant photoreceptors. Flies were reared in the dark and fixed at late pupal stages within 3 min after transfer to light conditions in order to avoid light-dependent Rh1 endocytosis. (A) A wild type ommatidium and (B–D) whole mutant ommatidia with only mutant photoreceptors from mosaic retinas. The genotypes are as follows: Rab6^546P^/Rab6^546P^ (B), Rab6^D23D^/Rab6 ^D23D^ (C) and Rich^1^/Rich^1^ (D). Inter-rhabdomeric spaces and MVBs are shown in purple and blue, respectively. (E) Wild type Golgi units and (F–H) mutant Golgi units with MVBs. The genotypes are Rab6^D23D^ (E, G) and Rich^1^ (H). (I) Quantification of MVBs in cross-sections of wild type, Rab6^546P^, and Rich^1^ mutant photoreceptors. MVBs were defined as membranous organelles with more than 4 inner vesicles larger than 300 nm in diameter; those 300–600 and ≥600 nm in diameter were classified as small and large, respectively. For quantification, photographs were taken randomly from the partial rescue Rab6^546P^ whole-eye clones or Rich^1^ whole-eye clones. Scale bars: 5 μm (A–D), 200 nm (E–H).

To investigate the details of organelle structures and plasma membrane domains, we observed thin sections of late pupal wild type, Rab6^546P^, Rab6^D23D^, and Rich^1^ whole mutant ommatidia by electron microscopy (Fig 5A–5D and S7 Fig). A tangential section of a wild type ommatidium contains 7 round rhabdomeres separated by the IRS. However, Rab6^546P^, Rich^1^, and Rab6^D23D^ ommatidia contained miniature rhabdomeres attached to each other, and a narrow IRS (Fig 5, purple) did not separate adjacent rhabdomere, similar to Eys^395^ and Eys^1^ mutants [4, 5]. The stalk membranes in Rab6^546P^, Rab6^D23D^ and Rich^1^ photoreceptors were shorter than those of the wild type, whereas the basolateral membranes were normal (S5G–S5I Fig). Similar to the confocal microscopic observations, these phenotypes were less prominent in Rich^1^ ommatidia than Rab6^546P^ and Rab6^D23D^ ommatidia. Overall, these plasma membrane morphological phenotypes are consistent with the immunohistochemically detected transport phenotypes of Rab6 and Rich mutants.

The appearance of the Golgi units in Rab6^546P^, Rab6^D23D^, and Rich^1^ photoreceptors also differed from those in the wild type: both Rab6- and Rich-deficient Golgi units were larger and well developed, and their cisternae were dilated (Fig 5E–5H), similar to previous reports [21–23]. However, in contrast to the report of Storrie et al., (21) the vesicle or budding profiles of Rab6 and Rich-deficient Golgi units were not obviously increased. In addition to the differences in these plasma membrane domains, the IRS, and Golgi units, the number and size of multi-vesicular bodies (MVBs) were greater in Rab6^546P^, Rab6^D23D^, and Rich^1^ photoreceptors than the wild type (Fig 5A–5D, blue). We quantified the MVBs in dark-adapted homozygous wild type, Rab6^546P^, and Rich^1^ photoreceptors, because MVB formation is strongly triggered by light-dependent Rh1 endocytosis even in wild type photoreceptors (Fig 5I). MVBs 300–600 and ≥600 nm in diameter were categorized as small and large, respectively. Adult wild type photoreceptors contained (mean ± SD) 0.15 ± 0.06 small MVBs and zero large MVBs. On the other hand, single Rab6^546P^ photoreceptors contained 0.33 ± 0.11 and 0.27 ± 0.09 small and large MVBs, respectively. Similarly, Rich^1^ photoreceptors contained 0.35 ± 0.09 and 0.27 ± 0.06 small and large MVBs, respectively. In addition to the increased number and size of MVBs, MVBs in Rab6- and Rich-deficient photoreceptors appeared different from those in the wild type photoreceptors; Rab6^546P^, Rab6^D23D^ and Rich^1^ photoreceptors contained many MVBs densely packed with inner vesicles that appeared partially degraded and were often accompanied by cisternae, some of which were Golgi units (Fig 5E–5H). The consistent sizes and populations of MVBs suggest that MVBs are likely identical to the Rab7-positive undefined cytoplasmic structures that accumulate Rh1, Eys, TRP and Chp in the 546P mutant (Figs 2 and 3).

### Rab6 localizes at the TGN and Golgi-associated Rab11-positive puncta

**Fig 6:**
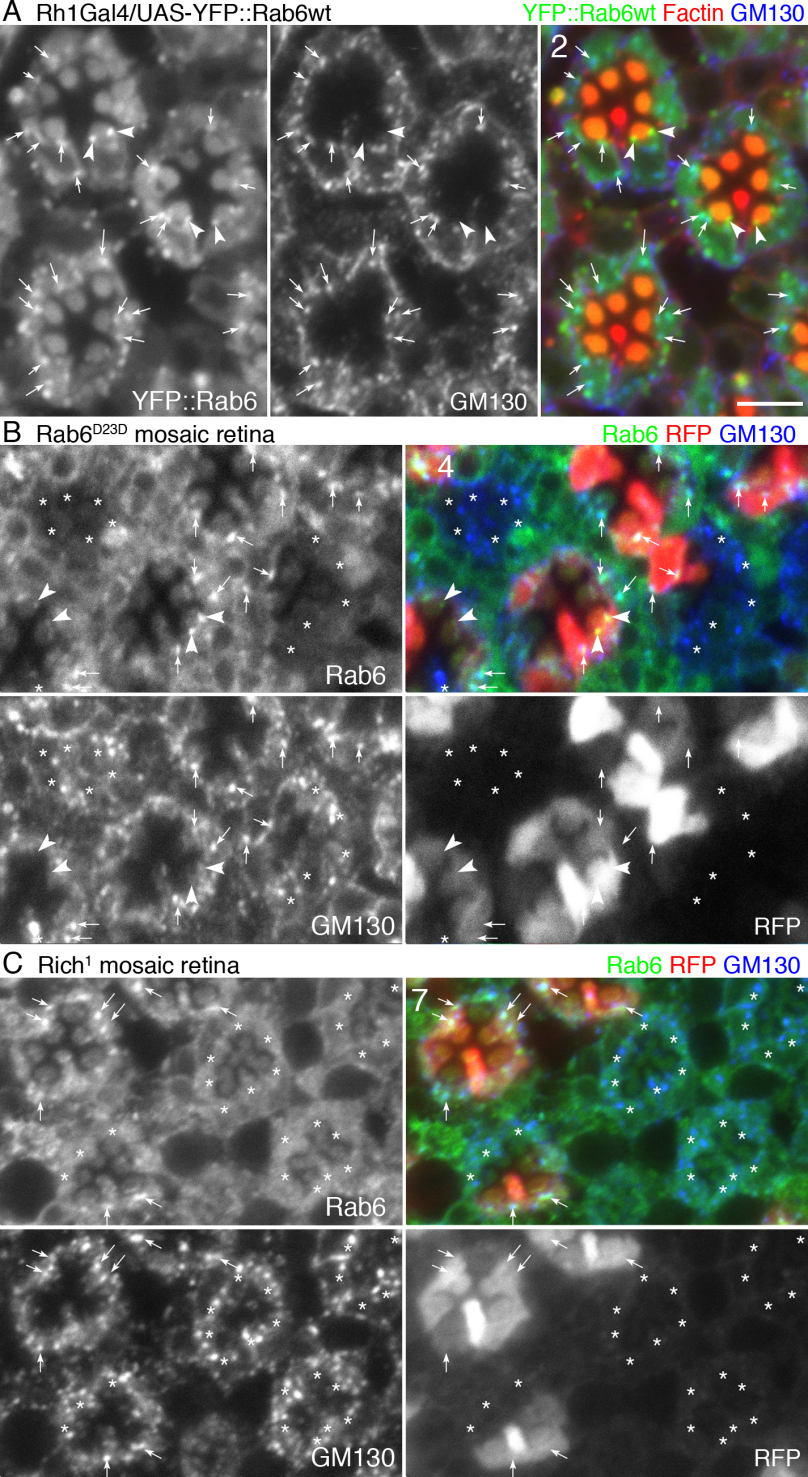
Rab6 localizes on Golgi and post-Golgi vesicles. (A) Immunostaining of wild type eyes expressing YFP::Rab6wt driven by Rh1-Gal4 stained with anti-GM130 (blue) and phalloidin (red). YFP::Rab6wt is shown in green. Significant autofluorescence was also observed in pigment granules in the green channel. (B) Rab6^D23D^ mutant mosaic eye immunostained by anti-Rab6 (GP1) (green) and anti-GM130 antibodies (blue). RFP (red) indicates wild type cells. Asterisks show Rab6^D23D^ mutant photoreceptors. (C) Rich^1^ mutant mosaic eye immunostained by anti-Rab6 (GP1) (green) and anti-GM130 antibodies (blue). RFP (red) indicates wild type cells. Asterisks show Rich^1^ mutant photoreceptors. Scale bars: 5 μm (A–C). Numbers of the samples observed were shown in the top-left corner of the composite images.

Further understanding the function of Rab6 requires knowing the localization of Rab6 protein in fly photoreceptors. In our first attempt, we used UAS-YFP::Rab6wt transgenic flies [34]. YFP::Rab6wt expressed by Rh1-Gal4 forms cytoplasmic foci in R1–6 photoreceptors. Most of the YFP::Rab6wt foci were associated with the *cis*-Golgi marker GM130 (Fig 6A, arrows), indicating these foci are located in Golgi units. GM130-negative YFP::Rab6wt foci at the base of the rhabdomeres were presumably post-Golgi vesicles bearing Rh1 (Fig 6A, arrowheads). Dispersed YFP signals were also observed in the cytoplasm and the rhabdomeres. The cytoplasmic YFP signal likely reflects GDP-bound form of YFP::Rab6wt associating with GDI. However, the reason for the YFP signal in the rhabdomeres is unclear; Rab6 might associate with some rhabdomeric proteins.

To confirm the localization of endogenous Rab6, we generated antisera against histidine-tagged full-length Rab6 protein in 1 rabbit and 2 guinea pigs (Rb anti-Rab6, GP1 anti-Rab6 and GP2 anti-Rab6). Western blotting showed that all antisera recognized a band around 22 kD in wild type head extract as well as a band around 60 kD in genomic Rab6^EYFP^ heterozygous head extracts (S8A Fig). Immunohistochemistry showed cytoplasmic foci only in wild type photoreceptors in Rab6^D23D^ mosaic retinas (Fig 6B, arrows and S8B Fig). Hence, these antisera specifically recognize Rab6. Rab6-positive cytoplasmic foci are also GM130-positive, suggesting these foci are Golgi units. Similar to YFP::Rab6wt, there were Rab6 foci at the bases of the rhabdomeres, which were GM130-negative, suggesting Rab6 also localizes on post-Golgi vesicles (Fig 6B, arrowheads). In Rich^1^ homozygous photoreceptors (Fig 6C, arrows), cytoplasmic Rab6-positive foci were not observed, suggesting that Rich is required for Rab6 recruitment to the Golgi membrane.

**Fig 7:**
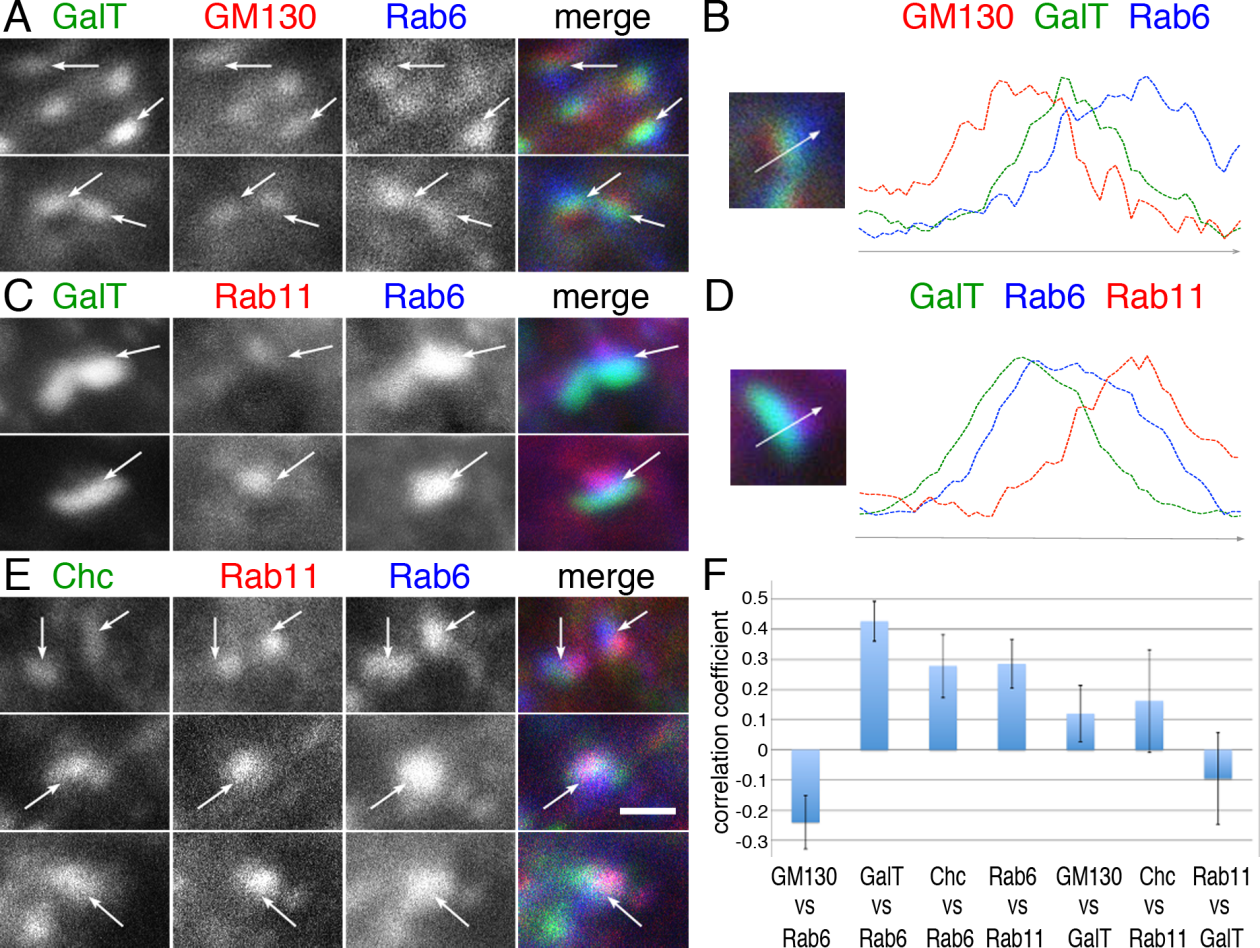
Rab6 localizes at the *trans*-cisternae/*trans*-Golgi network and Rab11-positive compartment. (A) Immunostaining of wild type eye expressing CFP-GalT (green) driven by GMR-Gal4 stained with anti-GM130 (red) and anti-Rab6 (GP1) (blue). (B) Intensity plots of signal intensity (y-axis) versus distance in mm (x-axis) show the occurrence of overlap between channels. Respective signals from the *cis*-Golgi markers GM130 (red), *trans*-Golgi marker CFP-GalT (green) and Rab6 (blue). (C) Immunostaining of wild type eye expressing CFP-GalT (green) driven by GMR-Gal4 stained with anti-Rab11 (red) and anti-Rab6 (GP1) (blue). (D) Intensity plots of signal intensity (y-axis) versus distance in mm (x-axis) show the occurrence of overlap between channels. Respective signals from the CFP-GalT (green), Rab6 (blue), and recycling endosome marker Rab11 (red). (E) Immunostaining of wild type eye by anti-Chc (green), anti-Rab11 (red), and anti-Rab6 (GP1) (blue). (F) Pearson’s correlation coefficients of the co-localization of the indicated protein sets. Scale bars: 1 μm (A, C, E).

Photoreceptors at earlier stages (i.e., ~20–30% pupal development) contain well-developed Golgi units, often reaching >1 μm in width [10]. To investigate the Golgi–cisternal association of Rab6, we used these large Golgi units in young pupal photoreceptors expressing a CFP-GalT marker, i.e., a cyan fluorescent protein fused to the 81 N-terminal amino acids of human β-1,4-galactosyltransferase [10], which localizes at the *trans*-cisternae of the Golgi units and the TGN [35, 36]. Double staining of CFP-GalT–expressing retina with anti-Rab6 and anti-GM130 showed that Rab6 localized to the opposite side of CFP-GalT–positive compartment from the *cis*-Golgi marker GM130 (Fig 7A). Detailed observations indicate the Rab6-positive region includes the TGN and extends more distally (Fig 7C).

Most of the Golgi units were accompanied by Rab11-positive puncta adjacent to the *trans* side (S9 Fig). Unlike the late pupal stages, when Rh1 transport is active and many Rab11-positive puncta localize at the base of the rhabdomeres, there are only a few Rab11-positive puncta apart from Golgi units in the young pupal stages. As Rab11 is typically considered as RE marker and is close to the TGN, these results suggest that these Rab11-positive puncta are likely fly photoreceptor REs emerging from the TGN or transport vesicles going to REs. Double staining of a CFP-GalT–expressing retina with anti-Rab6 and anti-Rab11 showed that Rab6 spreads from the CFP-GalT–positive TGN to the Golgi-associated Rab11-positive recycling endosomes (Figs 7B and S9 [wide view]). These results suggest that Rab6 regulates transport between the TGN and RE.

The Clathrin heavy chain (Chc) is an essential coat protein of some post-Golgi vesicles, especially for the basolateral pathway in epithelial cells [37]. Therefore, we compared Rab6 localization with Chc by triple staining wild type retinas with anti-Rab6, anti-Rab11, and anti-Chc (Fig 7C) [38]. Interestingly, Rab6-signals overlapped with both Rab11 and Chc, whereas Chc and Rab11 are largely separate (Fig 7C, upper panel). These results suggest that Rab6 functions in a step close to but before Rab11-dependent post-Golgi trafficking.

## Discussion

In the present study, screening for the failure of Rh1 accumulation in *Drosophila* photoreceptor rhabdomeres led to the re-identification of Rab6 as an essential molecule for Rh1 transport. Our detailed observations of Rh1 trafficking by BLICS indicate the inhibition of transport in Rab6-null mutants occurs within the Golgi units: Rh1 is transported to the *cis*-Golgi with normal kinetics but does not exit the Golgi units into the plasma membrane. Consequently, Rh1 greatly accumulates on the Golgi membrane and finally exits directly into the endosomal compartment. Furthermore, in Rab6 mutant photoreceptors, other rhabdomere membrane proteins (TRP and Chp), a stalk membrane protein (Crb), and the apically secreted protein (Eys) are reduced and degraded in MVBs together with Rh1. In contrast, the basolateral membrane proteins Na^+^K^+^ATPase and DE-Cad are unaffected. Moreover, Rab6 proteins are distributed from the TGN to Golgi-associated Rab11-positive structures.

Many aspects of Rab6 functions have been reported, such as retrograde transport from endosomes to the TGN, intra-Golgi or Golgi to ER trafficking, anterograde transport from the TGN, Golgi homeostasis, and Golgi-ribbon organization [39]. Nevertheless, there is no concrete understanding of all Rab6 functions. Among them, the defective morphology of the Golgi units and the failure of Rh1 transport observed in the present study are consistent with the results of Rab6 knockdown in HeLa cells by Storrie et al. [21], who report amplification and dilation of Golgi stacks, accumulation of unreleased vesicles on the *trans*-Golgi, inhibition of Golgi–plasma membrane transport of VSV-G without delayed ER–Golgi transport, and increased MVBs around the Golgi. The present results are also concordant with a study by Januschke et al., who report that mutants of Rab6b and a Rab6 GEF BicD in *Drosophila* oocytes induce accumulation of the TGF-α homolog Gurken in “ring-like particles” that have the properties of endolysosomes [40]. Two recent reports on HSV1 production and TNF secretion indicate Rab6 depletion inhibits the post-Golgi transport of HSV1 envelope proteins and TNF, respectively [22, 23]; these proteins accumulate at a juxtanuclear Golgi-like location and Golgi membranes in Rab6-depleted cells, respectively. These results are concordant with Rh1 localization after BLICS in our studies. Thus, together with the Rab6 localization at the *trans*-Golgi, the present results indicate that Rab6 is crucial for apical cargo export from the TGN.

The constitutively active GTP-locked form of Rab6 overexpressed by Rh1-promoter drastically reduces Rh1 content and retains Rh1 in the ER form in *Drosophila* photoreceptors [12]. Although the observed phenotype appears specific to rhodopsin and deficiency of ER–Golgi transport rather than post-Golgi transport, the phenotype is different from that in the present study likely because of differences in the methods used: Eys, TRP, and Crb expressions peak during the mid-pupal stage, whereas Rh1-promoter begins to be expressed at the late pupal stage. Gain or loss of Rab6 function causes defects in protein transport via the Golgi; however, they have opposite effects on the structure of Golgi units. Constitutively active Rab6 forces Golgi proteins to relocate to the ER [41] in contrast to the accumulation of the Golgi cisternae in Rab6-knockdown conditions [21]. Therefore, Rh1 may remain in the ER in the Rab6 hypermorph but stall at the TGN in the Rab6 amorph.

The results of the present study show that the Rab6-binding protein Rich is involved in rhabdomere and stalk membrane transport. Although its biochemical activity as a Rab6 GEF is unconfirmed [33], the loss of Rab6 localization to the Golgi in Rich^1^ mutant indicates Rich is required to recruit Rab6 to the Golgi membrane and likely works as a Rab6GEF like yeast and mammalian Ric1 [31, 32]. Rich was originally identified as a regulator of DN-Cad trafficking to synapses; Rab6 is also involved in these processes [33]. The present and previous findings collectively suggest Rab6/Rich-dependent export from the TGN is required for the trafficking of some axonal proteins as well as rhabdomeres and stalk proteins.

The most important finding of the present study is that in Rab6 mutants, the transport of protein cargos destined for the 2 apical membranes (i.e., the rhabdomere and stalk) is arrested and they accumulate together in the MVBs, while the basolateral cargos are transported normally. Hence, Golgi-localized Rab6 is not required for all plasma membrane-directed transport pathways. These results have implications for previous work on the *Drosophila* germline cyst [42] suggesting the existence of Rab6-dependent and Rab6-independent exocytic pathways. Although the roles of these multiple exocytic pathways in the polarized membrane domains are not mentioned, the Rab6-independent pathway to the plasma membrane in the germline cyst might correspond to the basolateral transport in photoreceptors. In addition, in embryonic salivary glands, Rab6 and Rab11 are localized on cytoplasmic vesicles containing an overexpressed apical membrane protein Cadherin 99C (Cad99C) [43]. Moreover, there is phenotypic similarity between Rab6 null and sec5 null. These findings indicate Rab6 and Rab11 sequentially regulate the apical transport of Cad99C, similar to Rh1 transport. However, in this case, as basolaterally transported truncated Cad99D also associates with Rab6, Rab6 and Cad99C colocalization merely reflects the association of Cad99C with Golgi units rather than apical transport vesicles.

Clathrin and AP1B are involved in basolateral transport [44, 45] and are required to sort various apical proteins in both mice and *C*. *elegans* [46–49]. In fly photoreceptors, the sole fly AP1 is essential for Na^+^K^+^ATPase transport towards the basolateral membrane, and the loss of the AP1 subunit, gamma, or mu causes mistransport of Na^+^K^+^ATPase to the stalk membrane [1], similar to zebrafish hair cells lacking AP1β subunits [50]. Clathrin likely plays a role in Na^+^K^+^ATPase transport towards the basolateral membrane in fly photoreceptors. The partial colocalization of Chc and Rab6 as well as the separation of Chc and Rab11 at the distal TGN (Fig 7B and 7D) indicate that the basolateral transport pathway likely branches off the Rab6-dependent apical transport pathway at the TGN. We previously showed that Rh1 transport from the TGN to the rhabdomere is dependent on Rab11 [10]. In addition, Rab6 localization is not restricted to the TGN but extends further to the Rab11-positive puncta closely associated with the TGN. These results imply that Rab6 is involved in Rh1 transport from the TGN to the closely associated Rab11-positive puncta. As Rab11 is commonly considered as RE marker, these TGN-associated Rab11-positive puncta are likely REs emerging from the TGN or transport vesicles going to REs. These results collectively suggest Rab6 regulates the trafficking of apical cargos from the TGN to REs in fly photoreceptors.

**Fig 8:**
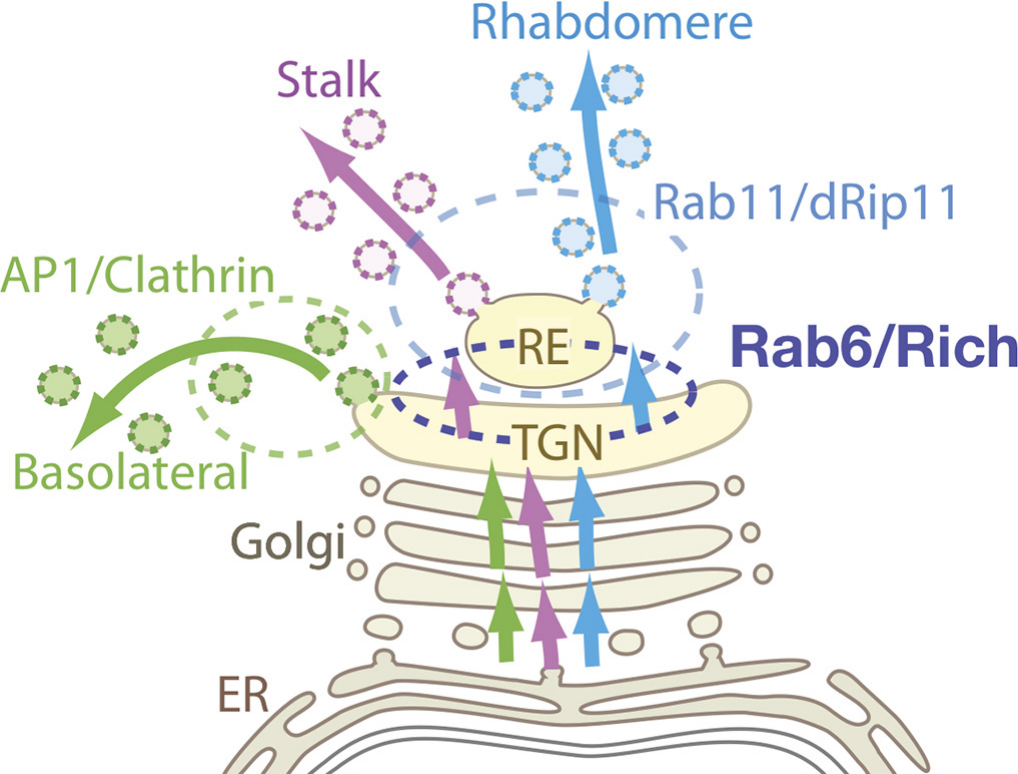
Proposed model of Rab6 function and 3 polarized transport pathways. Membrane proteins going to distinct plasma membrane domains are synthesized in the endoplasmic reticulum and transported together to the Golgi units. The first sorting event occurs within the TGN: the basolateral cargos are sorted into Chc/AP1 vesicles. Apical cargos bound for the rhabdomere or stalk membrane are not segregated within the TGN but are rather transported together to recycling endosomes by the Rab6-dependent process. The second sorting event at the recycling endosome segregates the cargos to the rhabdomere and stalk membrane, and carrier vesicles bearing the rhabdomere proteins are exported in a Rab11/dRip11/MyoV-dependent manner.

The proposed model of polarized transport in *Drosophila* photoreceptors is illustrated in Fig 8. Membrane proteins are synthesized on the ER and transported together to the Golgi units. Within the Golgi units, the basolateral cargos are sorted into Chc/AP1 vesicles at the TGN, whereas apical cargos bound for the rhabdomere or stalk membrane are not segregated within the TGN but are rather transported together to REs. Rab6 is essential for the TGN–RE transport of apical cargos. The second sorting event at the RE segregates the cargos to the rhabdomere and stalk membrane, and carrier vesicles bearing the rhabdomere proteins are exported in a Rab11/dRip11/MyoV-dependent manner.

The proposed model postulates the second sorting occurs at REs; however, this might occur earlier, for example at the TGN or between the TGN and REs. In addition, the direction of Rab6-mediated transport (i.e., anterograde or retrograde) remains unresolved, because anterograde cargo transport can be due to the retrograde recycling transport of the TGN-resident proteins. The mechanisms of intra-Golgi transport and molecular role of Rab6 remain controversial despite substantial research. Three models of transport between the TGN and REs are based on models of intra-Golgi transport. First, the vesicle transport model posits that Rab6 is involved in the anterograde transport of tubules/vesicles bearing apical cargos from the TGN to REs. Second, in the cisternal maturation model, the TGN gradually maturates into REs as TGN-resident proteins are retrieved by Rab6-dependent retrograde tubule/vesicle-mediated transport [51, 52]. Third, in the cisternal progenitor model, in which Rab GTPases including Rab6 define the identity of membrane subcompartments, continual fusion fission and the “Rab cascade” achieve cargo transport from the TGN to REs [53, 54]. Future studies should elucidate the molecular mechanisms by which Rab6 functions in the transport of apical cargos from the TGN to REs.

## Materials and Methods

### *Drosophila* stocks and genetic backgrounds

Flies were grown at 20–25°C on standard cornmeal–glucose–agar–yeast food unless indicated otherwise. Carotenoid-deprived food was prepared from 1% agarose, 10% dry-yeast, 10% sucrose, 0.02% cholesterol, 0.5% propionate, and 0.05% methyl 4-hydroxybenzoate.

EMS mutagenesis and F_1_ or F_2_ live-imaging screening were performed as described previously (27). Starter strain with the second chromosome carrying proximal neoFRT at 40A was isogenized from the Bloomington Stock 5615 (Bloomington, IN, USA), which is used in single nucleotide polymorphism (SNP) mapping [30].

The tester line w; Rh1Arr2GFP ey-FLP/TM6B were used for live imaging of mutant lines. Meanwhile, y w ey-FLP; P3RFP FRT40A/SM1 was used for immunostaining. Furthermore, y w 70FLP; Ubi-mRFP.nls FRT40A was used for the mosaic analysis of ovarian follicle cells.

To quantify MVBs by electron microscopy, mutant whole-eye clones were made for Rich^1^ using the EGUF/hid method [55]. For Rab6^546P^ and Rab6^D23D^, mutant whole-eye clones were not obtained probably because of the cell lethality of the mutants. Therefore, to partially rescue the lethality at early retinal development, YFP::Rab6wt was expressed by ey-Gal4, and Rab6^546P^ mutant whole-eye clones were obtained as described in the mutant analysis of Sec6 [11].

The following fly stocks were used: Rh1-Gal4, heat shock-Gal4, EGUF40A (Bloomington stock number 5250, Bloomington, IN, USA), UAS-YFP::Rab6wt (Bloomington stock number 23251, Bloomington, IN, USA), UAS-GFP::MyoVCT [9], Rab6^D23D^ FRT40A/CyO, Rich^1^ FRT80/TM3Kr>GFP, Rich^2^ FRT80/TM3, Kr>GFP (from Dr. Bellen, Baylor College of Medicine), and YRab6 (from Dr. Brankatschk, Max Planck Institute).

### Live imaging of fluorescent proteins expressed in photoreceptors

Fluorescent proteins expressed in photoreceptors were imaged by the water-immersion technique as described previously [8]. Briefly, late pupae with GFP-positive RFP mosaic retina were attached to the slide glass using double-sided sticky tape, and the pupal cases around the heads were removed. The pupae were chilled on ice, embedded in 0.5% agarose, and observed using an FV1000 confocal microscope equipped with a LUMPlanFI water-immersion 40× objective (Olympus, Tokyo, Japan).

### Immunohistochemistry

Fixation and staining methods were performed as described previously by Satoh and Ready [56]. Primary antisera were as follows: rabbit anti-Rh1 (1:1000) [10], chicken anti-Rh1 (1:1000) [1], rabbit anti-GM130 (1:300) (Abcam, Cambridge, UK), rabbit anti-NinaA (1:300) (a gift from Dr. Zuker, Columbia University), mouse monoclonal anti Na^+^K^+^-ATPase alpha subunit (1:500 ascites) (DSHB, IA, USA), rat monoclonal anti DE-Cad (1:20 supernatant) (DSHB, IA, USA), mouse monoclonal anti-Notch (17.9C6) (1:10 supernatant) (DSHB, IA, USA), mouse monoclonal anti-FasIII (7G10) (1:10 supernatant) (DSHB, IA, USA), mouse monoclonal anti-Chp (24B10) (1:20 supernatant) (DSHB, IA, USA), rat anti-Crb (a gift from Dr. Tepass, University of Toronto), rabbit anti-TRP (a gift from Dr. Montell, Johns Hopkins University), rabbit anti-Rab7 (1:1000) (a gift from Dr. Nakamura, Kumamoto University, Kumamoto, Japan), mouse monoclonal anti-Eys (1:20 supernatant) (DSHB, IA, USA), chicken anti-GFP (1:1000) (Chemicon International Inc., Billerica, MA, USA), rabbit anti-Chc (1:500) (a gift from Dr. Kametaka, Nagoya University, Nagoya, Japan), mouse anti-Rab11 (1:250) [10], and rabbit anti-Rab11 (1:300) [10]. Secondary antibodies were anti-mouse, anti-rabbit, anti-rat and/or anti-chicken antibodies labelled with Alexa Fluor 488, 568, and 647 (1:300) (Life Technologies, Carlsbad, CA, USA) or Cy2 (1: 300) (GE Healthcare Life Sciences, Pittsburgh, PA, USA). Images of samples were recorded using an FV1000 confocal microscope (60× 1.42 NA objective lens; Olympus, Tokyo, Japan). To minimize bleed-through, each signal in double- or triple-stained samples was imaged sequentially. Images were processed in accordance with the Guidelines for Proper Digital Image Handling using ImageJ and/or Adobe Photoshop CS3 (Adobe, San Jose, CA, USA).

CFP was detected using the virtual channel function of the FV1000, which stacks CFP images taken by the other dichroic mirror on other channel images (i.e., Alexa Fluor 488, 568, and 647). However, the CFP laser beam axis was not perfectly aligned with the others probably because of the change in the dichroic mirror. Therefore, we scored this misalignment according to anti-GFP antibody staining of CFP with secondary antibodies conjugated with Alexa Fluor 488 and 647, and calibrated each of the 3 colored pictures obtained.

### BLICS

Newly eclosed flies fed carotenoid-deprived food were switched to carotenoid-deprived food with crystalline *all-trans* retinal (Sigma, St. Louis, MO, USA) in the dark. After 1 or 2 nights in the dark, the flies were irradiated with a 405-nm diode laser module at 30 mW for 20 min (Pepaless, Hyogo, Japan) to isomerize the *all-trans* retinal form to the 11-*cis* form and initiate Rh1 maturation.

### Electron microscopy

Electron microscopy was performed as described previously [8]. Samples were observed on a JEM1400 electron microscope (JEOL, Tokyo, Japan), and montage images were taken by a CCD camera system (JEOL, Tokyo, Japan).

MVBs were counted in the tangential sections of 12–36 photoreceptor cells of individual flies, and the means and standard deviations were calculated from 4 flies for each allele.

### Mapping and determination of mutations

Meiotic recombination mapping was performed according to the standard method [57]. Briefly, to allow meiotic recombination between the proximal FRT, the phenotype-responsible mutation, and a distal miniature w^+^ marker, flies carrying isogenized chromosome 546P were crossed with flies with isogenized P{EP0511}, which carry the miniature w^+^ marker near the distal end of arm 2L. Female offspring carrying the mutated chromosome and the miniature w^+^-marked chromosome were crossed with males carrying FRT40A and P3RFP on the isogenized second chromosome and Rh1Arr2GFP on the third chromosome. Live imaging was to judge whether the resultant adult offspring with w^+^ mosaic (i.e., maternally inherited FRT and w^+^) inherited the mutation responsible for the Rh1 transport defect. The recovered flies were individually digested in 50 μL 200 ng/μL proteinase K in 10mM Tris-Cl (pH 8.2), 1 mM EDTA, and 25 mM NaCl at 55°C for 1 h and subsequently heat inactivated at 85°C for 30 min and 95°C for 5 min. Then, 0.5 μL digested solution was used as the template for PCR amplification for RFLP analysis according to the method described in the FlySNP database ([58], http://flysnp.imp.ac.at/index.php). The mutation responsible for the Rh1 transport defect was mapped between SNP markers 893 and 977 defined in the FlySNP database.

### Whole-genome resequencing of EMS-generated mutants

For the whole-genome resequencing of the 546P mutant, the second chromosome was balanced over a balancer, CyO, P{Dfd-GMR-nvYFP}(Bloomington stock number 23230, Bloomington, IN, USA), to facilitate the isolation of homozygous embryo. Using the REPLI-G single-cell kit (QIAGEN, Hilden, Germany), the genomic DNAs were independently amplified from four 546P homozygous embryos. The pair-end library was prepared using the Nextera DNA sample prep kit (Illumina, San Diego, CA, USA) for each embryo, 2 × 250 bp reads were obtained using the MiSeq v2 kit (Illumina, San Diego, CA, USA). Reads were mapped to release 5 of the *D*. *melanogaster* genome using BWA 0.7.5a. The RFLP-mapped region of 546P was covered by reads with an average depth of 29.5x and width of 97.8%. Mapped reads were processed using Picard-tools 1.99 and the Genome Analysis Tool Kit 2.7–2 (GATK, Broad Institute, Cambridge, MA, USA). Single nucleotide variants and indels were called using Haplotypecaller in GATK, and those of the isogenized starter stock were subtracted to extract the unique variants in 546P and annotated using SnpSift [59] In the mapped region, there were 4 unique variants that could cause some defects in gene function (i.e., CG31705 P453L, Rab6 R73STOP, ACXE A193T, and nimB1 G255E). Complementation tests over defined chromosomal deletions covering the RFLP-mapped region (i.e., BSC213, BSC241, BSC242, BSC244, ED775, ED761, ED780, and ED791) revealed that only ED775 and ED761 were lethal to 546P homozygous larvae. The region deleted in both ED775 and ED761 contained the Rab6 R73STOP mutation in 546P, whereas this was not present in the other 3 variants described above.

The point mutation of Rab6 R73STOP on 2L:12108583 (Release 5) was verified by capillary sequencing of PCR-amplified fragment using the following primers: 5′-AGCGGAGCGAGAAGAGAGTT-3′ and 5′-GCTCCTGCTGTTGAAAAAGG-3′.

### Antisera against Rab6

Full-length cDNA encoding Rab6 was amplified from a cDNA clone isolated from the fly retina cDNA library [60] and cloned into *pQE60*. The 6xHis-tagged Rab6 protein was expressed in *E*. *coli* pG-KJE8/BL21 (TAKARA) at 23°C and purified in native conditions using Ni-NTA Agarose (QIAGEN, Hilden, Germany). To obtain antisera, 1 rabbit and 2 guinea pigs were immunized 6 times with 200 μg 6xHis-Rab6 protein (Hashimoto, Wakayama, Japan). We designated the resultant antisera Rb anti-Rab6, GP1 anti-Rab6, and GP2 anti-Rab6.

### Immunoblotting

Immunoblotting was performed as described previously [8]. The following antibodies were used: rabbit anti-Rab6 (1:2000 concentrated supernatant) (made in house), 2 guinea pig anti-Rab6 (1:2000 concentrated supernatant) (made in house) as primary antibodies; HRP-conjugated anti-rabbit or anti-guinea pig IgG antibodies (1:20,000, Life Technologies, Carlsbad, CA, USA) as a secondary antibody. Signals were visualized using enhanced chemiluminescence (Clarity Western ECL Substrate; Bio-Rad, Hercules, CA, USA) and imaged using ChemiDoc XRS+ (Bio-Rad, Hercules, CA, USA).

## Supporting Information

S1 Fig Rab7-positive endosomes localize adjacent to Golgi units 90 min after blue light-induced chromophore supply (BLICS). Eighteen slices of 546P mutant ommatidium at 0.5-μm intervals 90 min after BLICS were used for the projection image in Fig 3G. Rh1, Rab7 (endosome marker) and p120 (medium Golgi marker) are indicated green, red, and blue, respectively. Arrows indicate close localization of Rab7-positive endosomes to Golgi units. Rh1 localizes in both organelles. Scale bars: 5 μm
(TIF)

S2 Fig Rab6^D23D^ and Rich^1^ mutant photoreceptors exhibit defective Rh1 and Eys transport but not Na^+^K^+^ATPase transport. (A, B) Rab6^D23D^ mutant mosaic eye immunostained by phalloidin (green) and anti-Eys antibody (blue) (A) or anti-Na^+^K^+^ATPase (green) and anti-Rh1 (blue) antibodies (B). RFP (red) indicates wild type cells. Asterisks show Rab6^D23D^ mutant photoreceptors. (C, D) Rich^1^ mutant mosaic eyes immunostained by phalloidin (green) and anti-Eys antibody (blue) (C) or anti-Na^+^K^+^ATPase (green) and anti-Rh1 (blue) antibodies (D). RFP (red) indicates wild type cells. Asterisks show Rich^1^ mutant photoreceptors. Scale bars: 5 μm (A–D). Numbers of the samples observed were shown in the top-left corner of the composite images.
(TIF)

S3 Fig Kinetics of Rh1 transport in Rab6^D23D^ and Rich^1^ mutant mosaic eyes. Rab6^D23D^ (A–D) and Rich^1^ (E–H) mutant mosaic eyes immunostained by the indicated antibodies. RFP (red) mark wild type cells. Asterisks show Rab6^D23D^ or Rich^1^ homozygous photoreceptors. (A, E) Immunostaining before BLICS. Anti-Rh1 (green) and anti-NinaA antibodies (blue, endoplasmic reticulum marker). (B, F) Immunostaining 40 min after BLICS. Anti-Rh1 (green) and anti-GM130 (blue, Golgi marker) antibodies. (C, G) Immunostaining 90 min after BLICS. Anti-Rh1 (green) and anti-GM130 (blue, Golgi marker) antibodies. (D, H) Immunostaining 180 min after BLICS. Anti-Rh1 (green) and anti-Rab7 (blue, late endosome marker) antibodies. Scale bars: 5 μm (A–H). Numbers of the samples observed were shown in the top-left corner of the composite images.
(TIF)

S4 Fig Membrane protein localizations in Rab6^D23D^ and Rich^1^ mutant mosaic eyes. Rab6^D23D^ (A–C) and Rich^1^ (D–F) mutant mosaic eyes immunostained by the following antibodies or phalloidin. RFP (red) indicates wild type cells. Asterisks show Rab6^D23D^ or Rich^1^ homozygous photoreceptors. (A, D) Anti-Chp (green) and anti-Rh1 antibodies (blue). (B, E) Anti-Crb (green) and anti-Rh1 (green). (C, F) Anti-TRP (green) and anti-–DE-Cad antibodies (blue). Scale bars: 5 μm (A–F). Numbers of the samples observed were shown in the top-left corner of the composite images.
(TIF)

S5 Fig Quantification of basolateral and stalk membranes in wild type, Rab6^546P^, Rab6^D23D^, and Rich^1^ mutant photoreceptors. (A–C) The lengths of immunostained basolateral (B) and stalk membranes (C) by anti-Na^+^K^+^ATPase and anti-Crb antibodies as well as the ratio of their lengths (A) were measured in wild type, Rab6^546P^/Rab6^546P^, Rab6^D23D^/Rab6^D23D^ and Rich^1^/Rich^1^ photoreceptors. (D–F) The lengths of immunostained basolateral (B) and stalk membranes (C) on confocal microscopy as well as the ratio of their lengths (A) were measured in the above mentioned photoreceptors. (G–I) The lengths of basolateral (H) and stalk membranes (I) on electron microscopy as well as the ratio of their lengths (G) were measured in the above mentioned photoreceptors.
(TIF)

S6 Fig Localizations of apical and basolateral membrane proteins in Rab6^D23D^ mutant ovarian follicle cells. Immunostaining of a mosaic ovary containing both wild type and Rab6^D23D^/Rab6^D23D^ ovarian follicle cells by anti-FasIII (A, green) and anti-Notch (B, green). RFP (red) indicates wild type cells. Asterisks show Rab6^D23D^ homozygous follicle cells. Scale bars: 1 μm.
(TIF)

S7 Fig Uncolored pictures of Fig 5. (A) Wild type ommatidium and (B–D) whole mutant ommatidia with only mutant photoreceptors from mosaic retinas. The genotypes are as follows: Rab6^546P^/Rab6^546P^ (B), Rab6^D23D^/Rab6 ^D23D^ (C) and Rich^1^/Rich^1^ (D). Scale bars: 5 μm.
(TIF)

S8 Fig Three anti-Rab6 antisera recognize Rab6 according to immunoblotting and immunocytochemistry. (A) Immunoblotting of fly head extracts obtained from wild type and Rab6^EYFP^ heterozygous flies (Rab6^EYFP/+^) by using Rb anti-Rab6, GP1 anti-Rab6, and GP2 anti-Rab6 antisera. Rab6^EYFP^ expresses the endogenously tagged Rab6 protein, whose allele were generated by ends-in gene targeting [61]. Arrows indicate the bands corresponding to Rab6 and Rab6^EYFP^. (B) Rab6^D23D^ mutant mosaic eyes were immunostained by Rb anti-Rab6, GP1 anti-Rab6, and GP2 anti-Rab6 antisera. These mosaic eyes were counterstained by Alexa488-condjugated phalloidin to visualize the structure of photoreceptors. RFP (red) indicates wild type cells. Asterisks show Rab6^D23D^ homozygous photoreceptors. Scale bars: 5 μm (B). Numbers of the samples observed were shown in the top-left corner of the composite images.
(TIF)

S9 Fig Wide view of Rab6 localization at the *trans*-cisternae/*trans*-Golgi network and Rab11-positive compartment. Immunostaining of a wild type eye expressing CFP-GalT (green) driven by GMR-Gal4 with anti-Rab11 (red) and anti-Rab6 (GP1) (blue). Scale bars: 1 μm.
(TIF)
