# Supplementary figures and images for: Rab6 Is Required for Multiple Apical Transport Pathways but Not the Basolateral Transport Pathway in Drosophila Photoreceptors
Source: PLoS Genet. 2016 Feb 18;12(2):e1005828. doi: 10.1371/journal.pgen.1005828 (PMC4758697; doi:10.1371/journal.pgen.1005828)

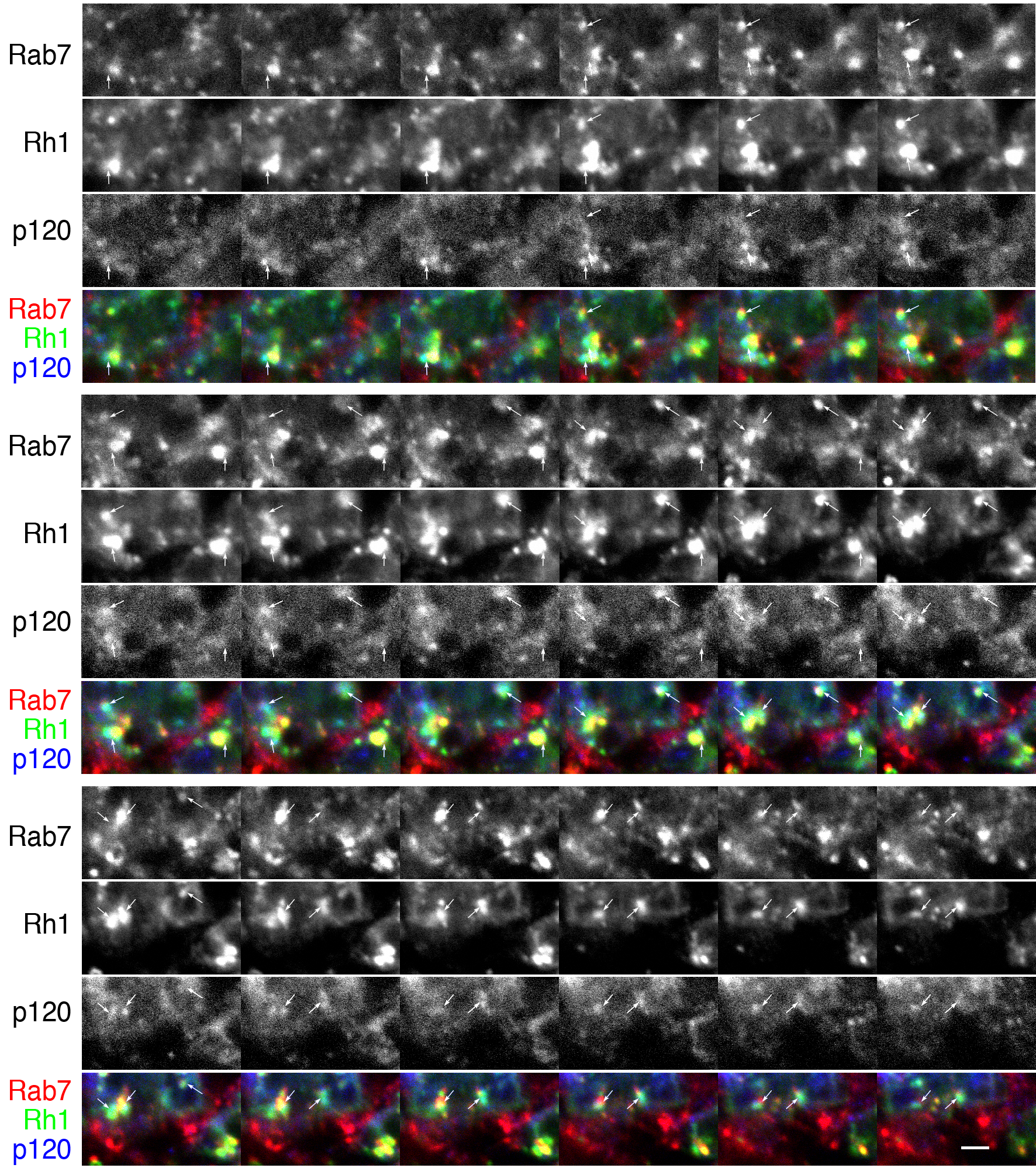

Supplement: S1 Fig — Eighteen slices of 546P mutant ommatidium at 0.5-μm intervals 90 min after BLICS were used for the projection image in Fig 3G. Rh1, Rab7 (endosome marker) and p120 (medium Golgi marker) are indicated green, red, and blue, respectively. Arrows indicate close localization of Rab7-positive endosomes to Golgi units. Rh1 localizes in both organelles. Scale bars: 5 μm (TIF) [file pgen.1005828.s001.tif]

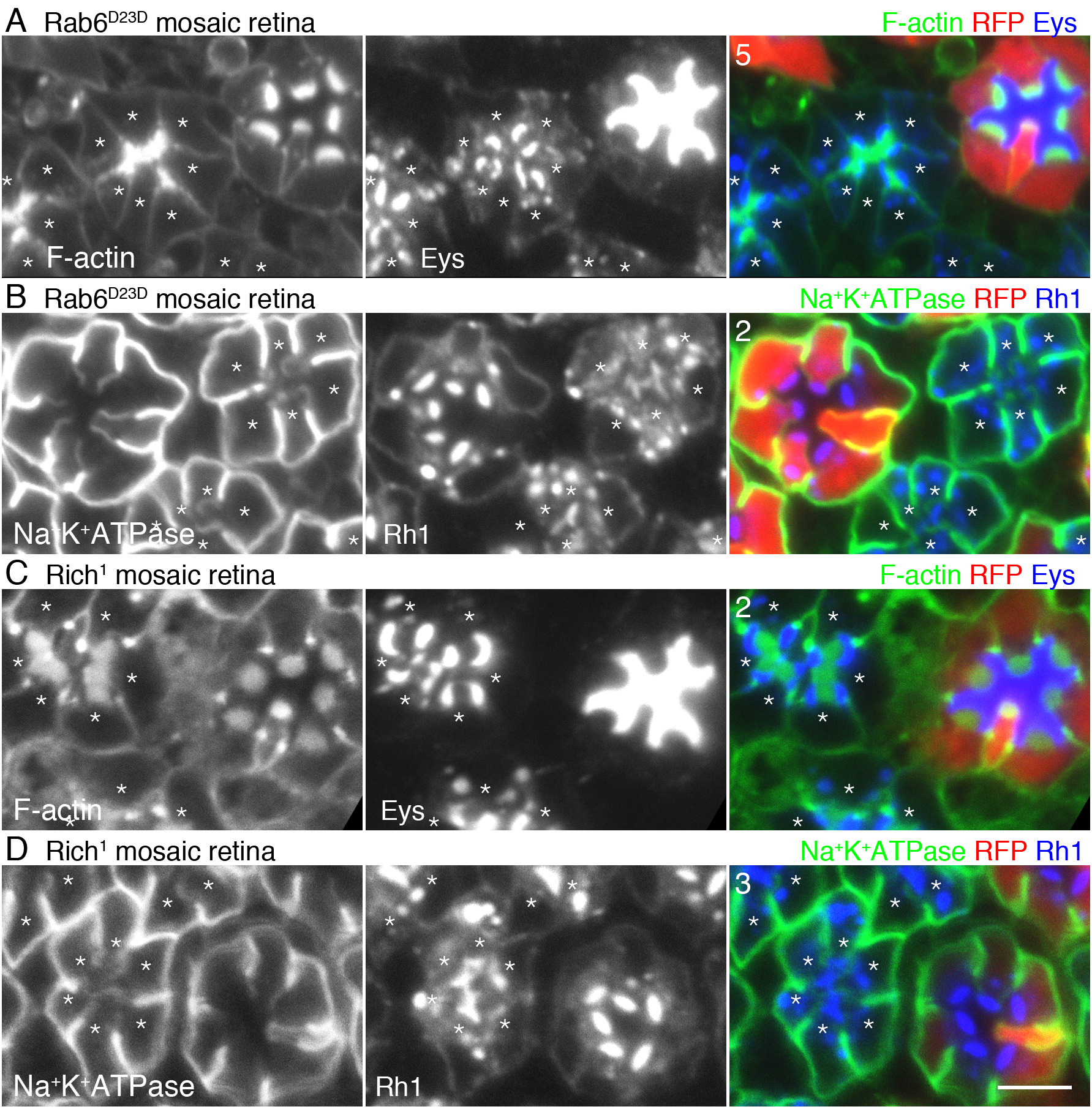

Supplement: S2 Fig — (A, B) Rab6D23D mutant mosaic eye immunostained by phalloidin (green) and anti-Eys antibody (blue) (A) or anti-Na+K+ATPase (green) and anti-Rh1 (blue) antibodies (B). RFP (red) indicates wild type cells. Asterisks show Rab6D23D mutant photoreceptors. (C, D) Rich1 mutant mosaic eyes immunostained by phalloidin (green) and anti-Eys antibody (blue) (C) or anti-Na+K+ATPase (green) and anti-Rh1 (blue) antibodies (D). RFP (red) indicates wild type cells. Asterisks show Rich1 mutant photoreceptors. Scale bars: 5 μm (A–D). Numbers of the samples observed were shown in the top-left corner of the composite images. (TIF) [file pgen.1005828.s002.tif]

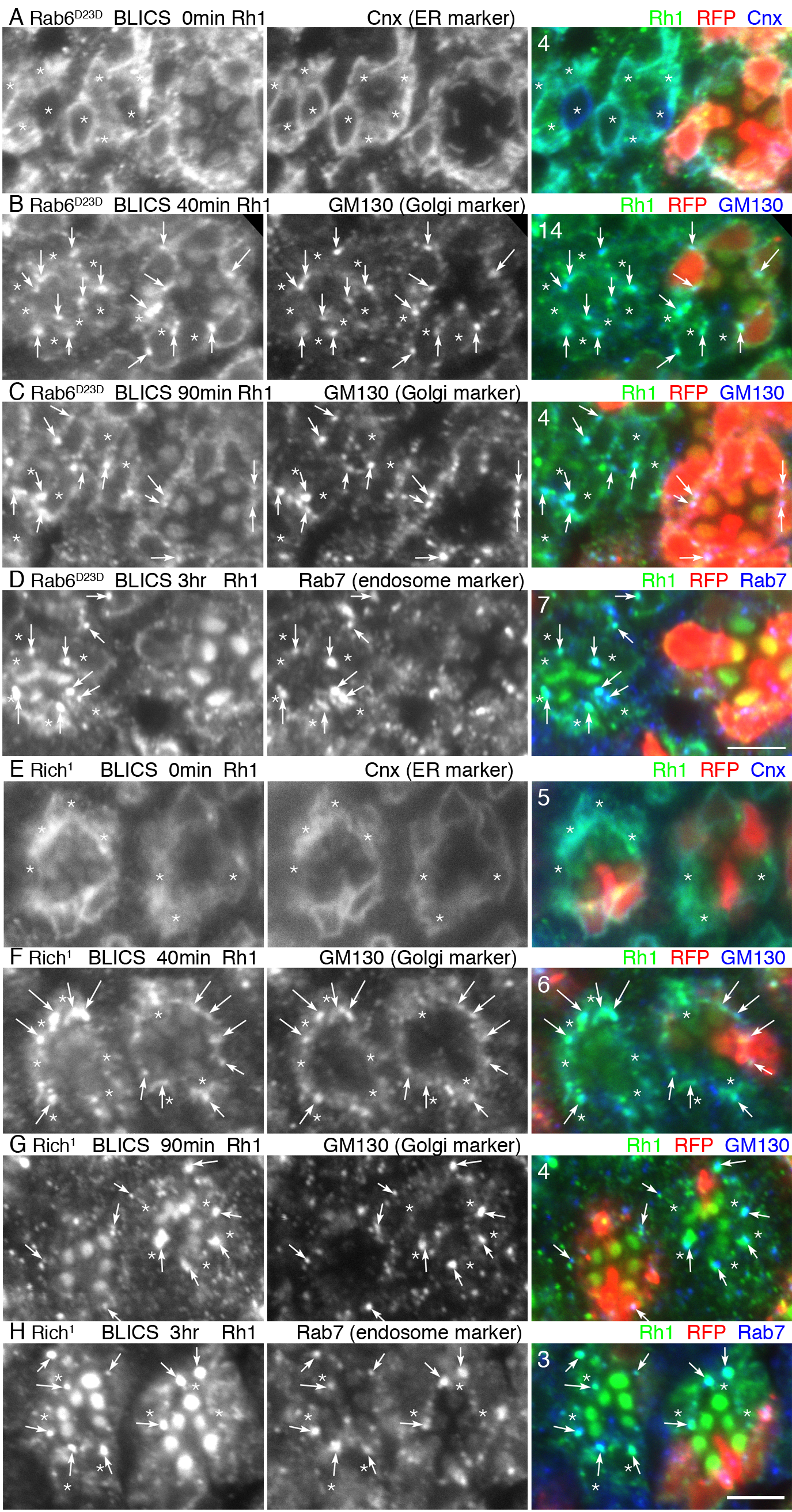

Supplement: S3 Fig — Rab6D23D (A–D) and Rich1 (E–H) mutant mosaic eyes immunostained by the indicated antibodies. RFP (red) mark wild type cells. Asterisks show Rab6D23D or Rich1 homozygous photoreceptors. (A, E) Immunostaining before BLICS. Anti-Rh1 (green) and anti-NinaA antibodies (blue, endoplasmic reticulum marker). (B, F) Immunostaining 40 min after BLICS. Anti-Rh1 (green) and anti-GM130 (blue, Golgi marker) antibodies. (C, G) Immunostaining 90 min after BLICS. Anti-Rh1 (green) and anti-GM130 (blue, Golgi marker) antibodies. (D, H) Immunostaining 180 min after BLICS. Anti-Rh1 (green) and anti-Rab7 (blue, late endosome marker) antibodies. Scale bars: 5 μm (A–H). Numbers of the samples observed were shown in the top-left corner of the composite images. (TIF) [file pgen.1005828.s003.tif]

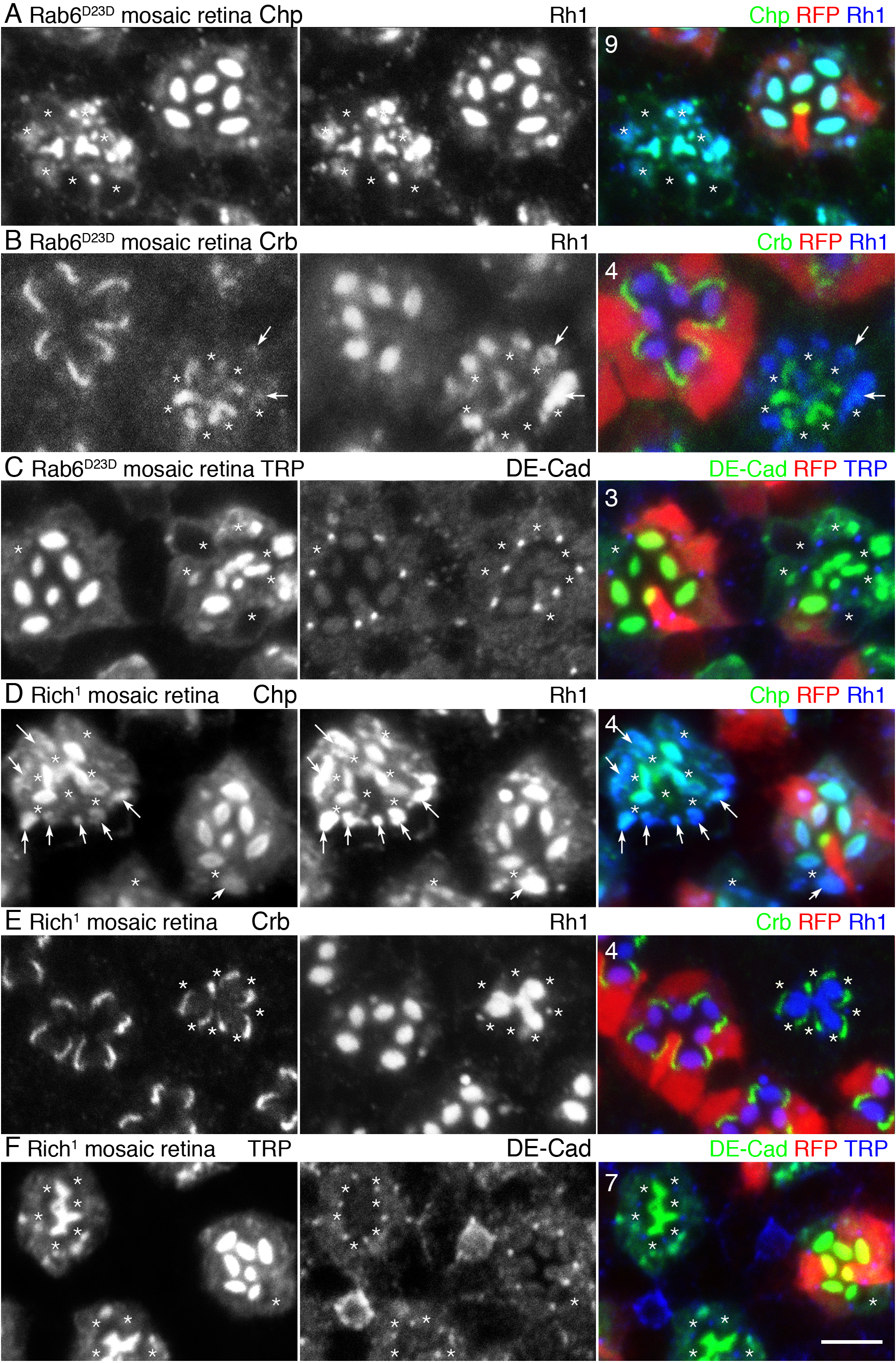

Supplement: S4 Fig — Rab6D23D (A–C) and Rich1 (D–F) mutant mosaic eyes immunostained by the following antibodies or phalloidin. RFP (red) indicates wild type cells. Asterisks show Rab6D23D or Rich1 homozygous photoreceptors. (A, D) Anti-Chp (green) and anti-Rh1 antibodies (blue). (B, E) Anti-Crb (green) and anti-Rh1 (green). (C, F) Anti-TRP (green) and anti-–DE-Cad antibodies (blue). Scale bars: 5 μm (A–F). Numbers of the samples observed were shown in the top-left corner of the composite images. (TIF) [file pgen.1005828.s004.tif]

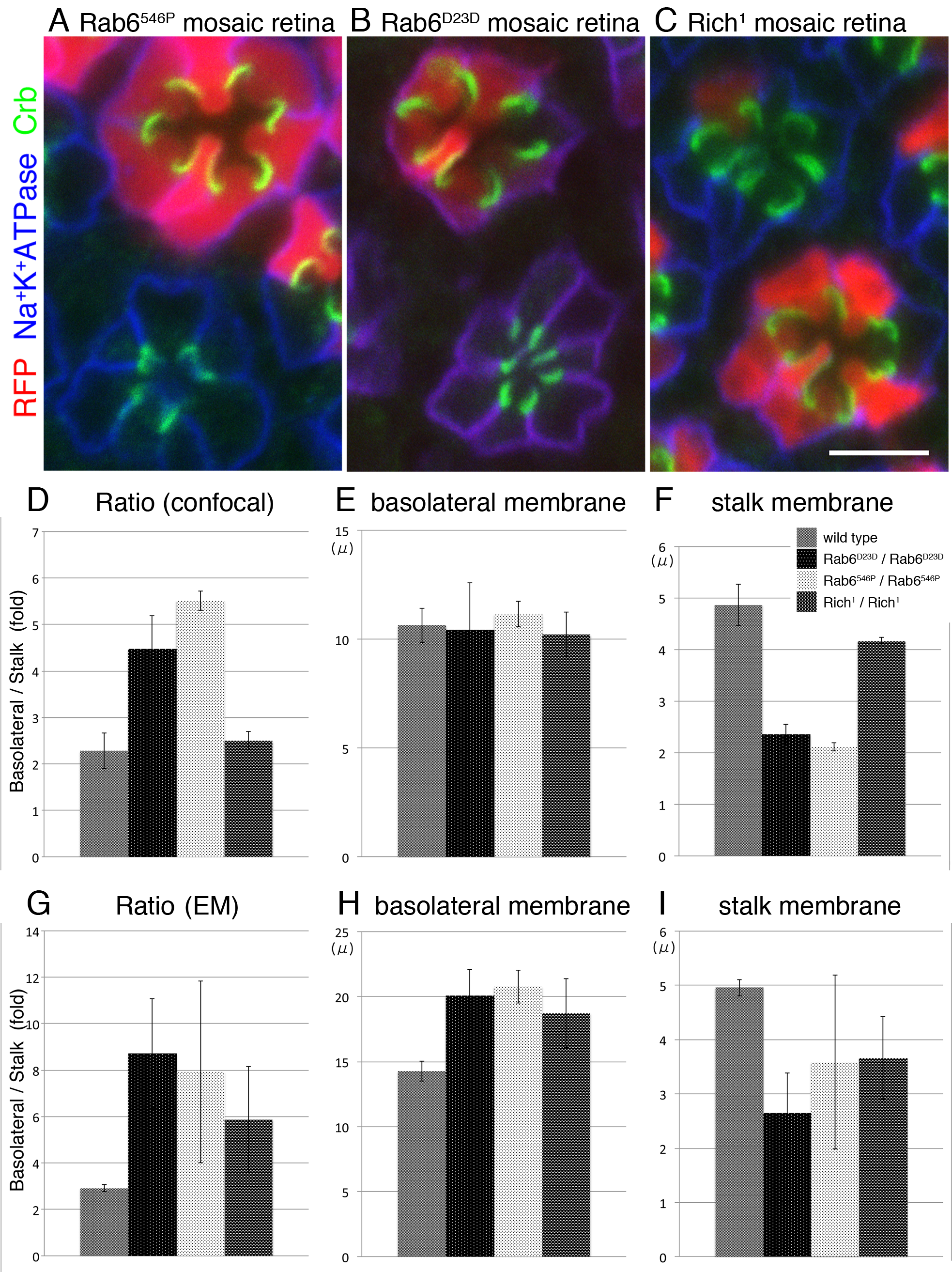

Supplement: S5 Fig — (A–C) The lengths of immunostained basolateral (B) and stalk membranes (C) by anti-Na+K+ATPase and anti-Crb antibodies as well as the ratio of their lengths (A) were measured in wild type, Rab6546P/Rab6546P, Rab6D23D/Rab6D23D and Rich1/Rich1 photoreceptors. (D–F) The lengths of immunostained basolateral (B) and stalk membranes (C) on confocal microscopy as well as the ratio of their lengths (A) were measured in the above mentioned photoreceptors. (G–I) The lengths of basolateral (H) and stalk membranes (I) on electron microscopy as well as the ratio of their lengths (G) were measured in the above mentioned photoreceptors. (TIF) [file pgen.1005828.s005.tif]

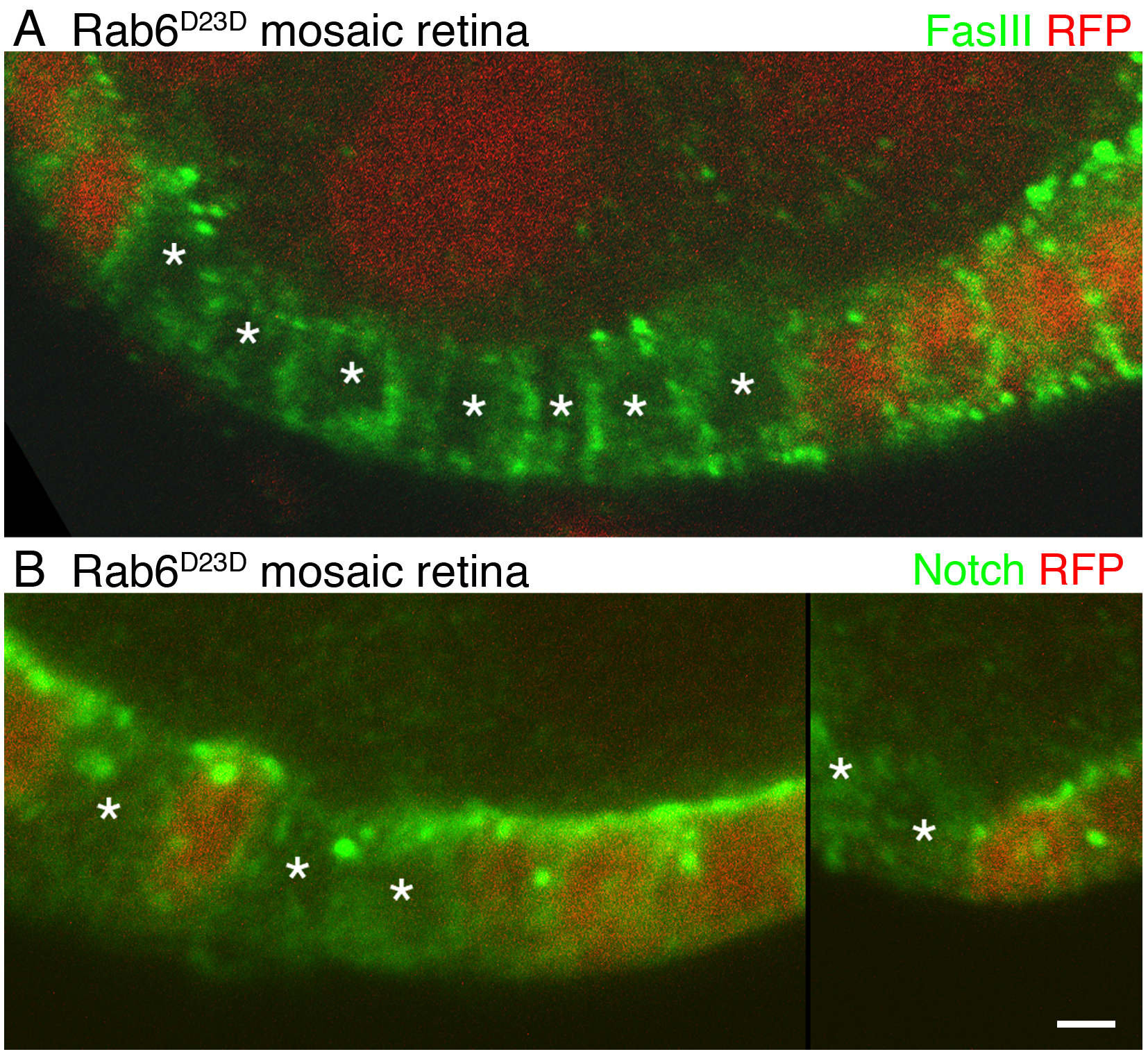

Supplement: S6 Fig — Immunostaining of a mosaic ovary containing both wild type and Rab6D23D/Rab6D23D ovarian follicle cells by anti-FasIII (A, green) and anti-Notch (B, green). RFP (red) indicates wild type cells. Asterisks show Rab6D23D homozygous follicle cells. Scale bars: 1 μm. (TIF) [file pgen.1005828.s006.tif]

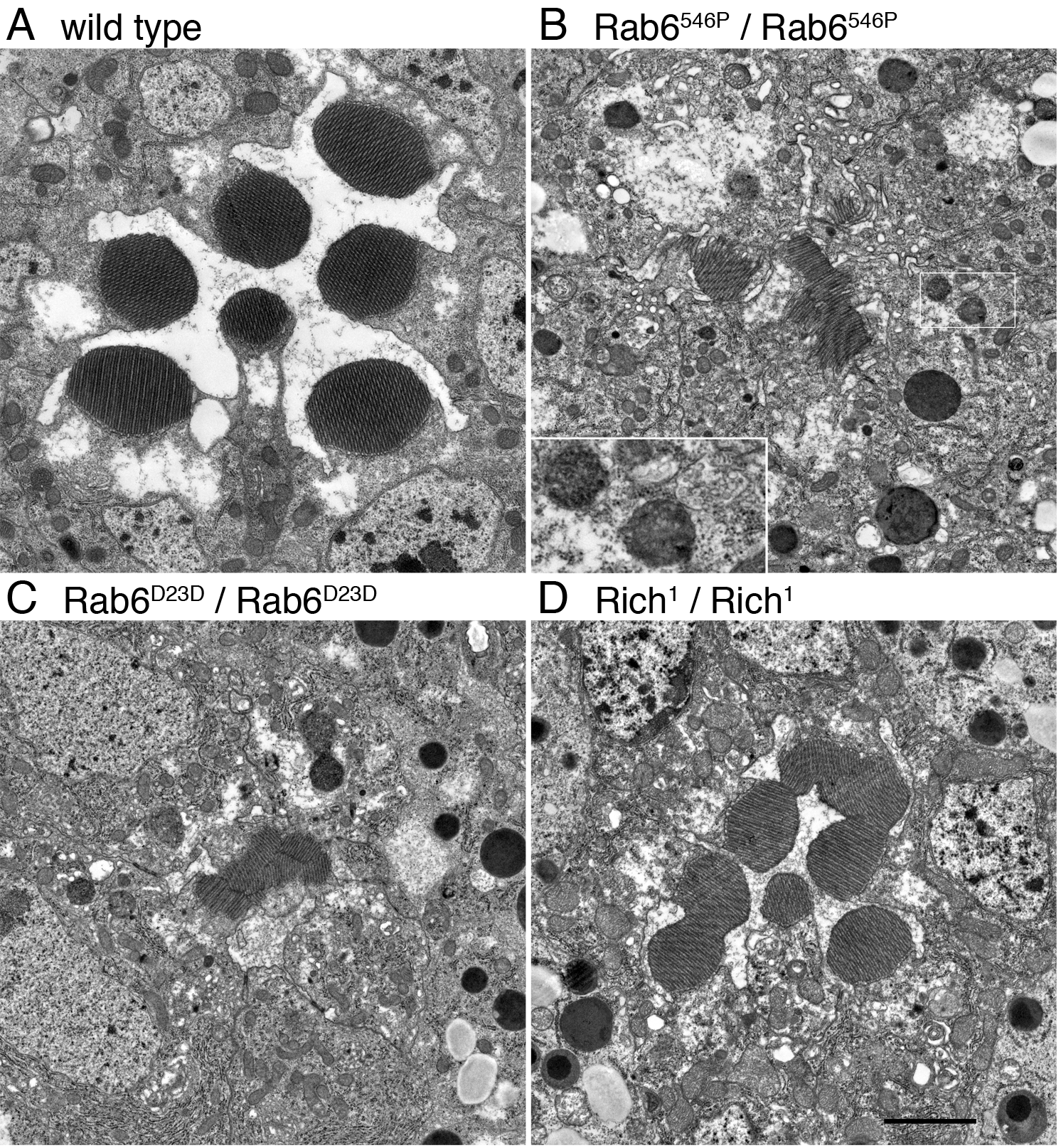

Supplement: S7 Fig — (A) Wild type ommatidium and (B–D) whole mutant ommatidia with only mutant photoreceptors from mosaic retinas. The genotypes are as follows: Rab6546P/Rab6546P (B), Rab6D23D/Rab6 D23D (C) and Rich1/Rich1 (D). Scale bars: 5 μm. (TIF) [file pgen.1005828.s007.tif]

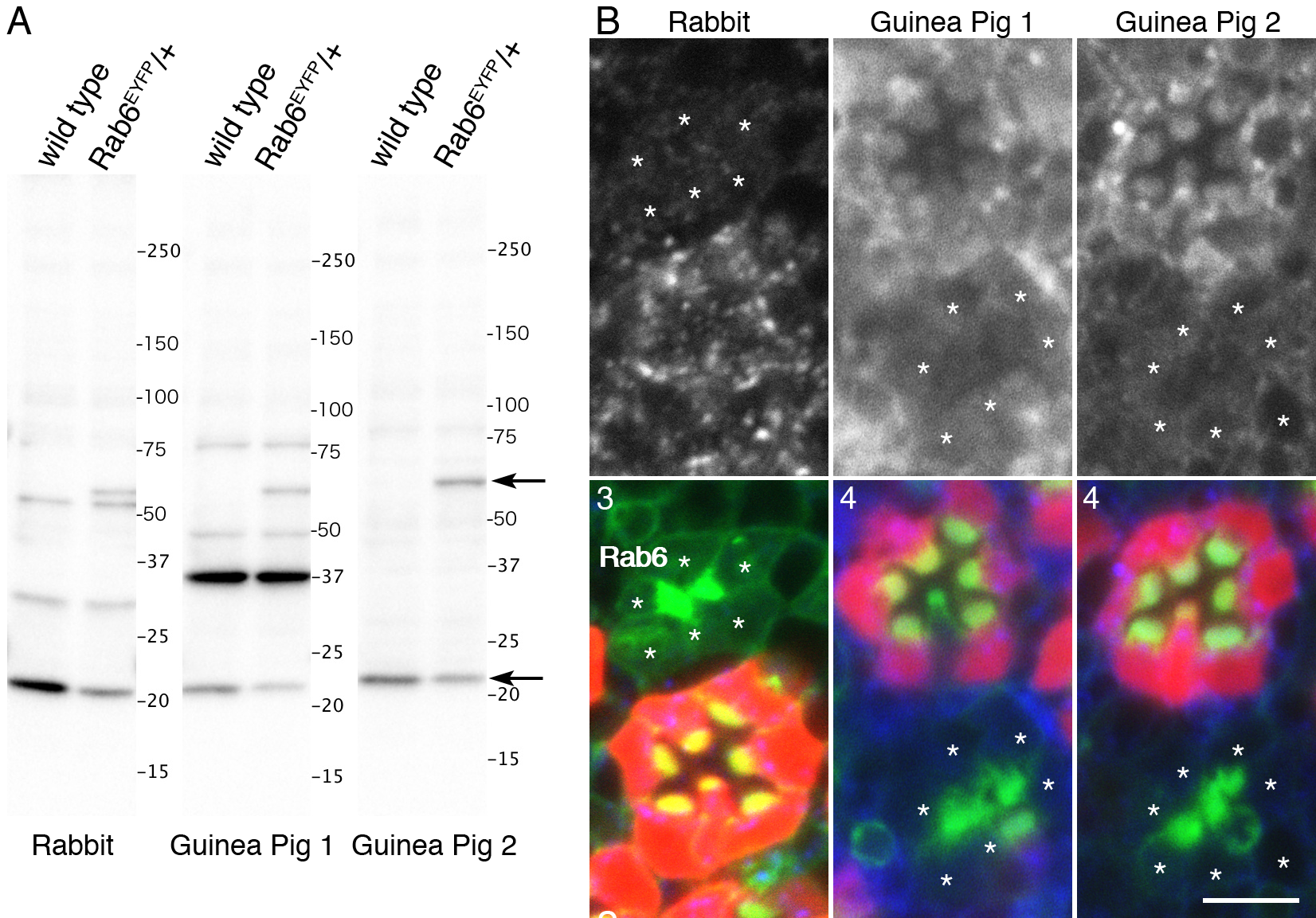

Supplement: S8 Fig — (A) Immunoblotting of fly head extracts obtained from wild type and Rab6EYFP heterozygous flies (Rab6EYFP/+) by using Rb anti-Rab6, GP1 anti-Rab6, and GP2 anti-Rab6 antisera. Rab6EYFP expresses the endogenously tagged Rab6 protein, whose allele were generated by ends-in gene targeting [61]. Arrows indicate the bands corresponding to Rab6 and Rab6EYFP. (B) Rab6D23D mutant mosaic eyes were immunostained by Rb anti-Rab6, GP1 anti-Rab6, and GP2 anti-Rab6 antisera. These mosaic eyes were counterstained by Alexa488-condjugated phalloidin to visualize the structure of photoreceptors. RFP (red) indicates wild type cells. Asterisks show Rab6D23D homozygous photoreceptors. Scale bars: 5 μm (B). Numbers of the samples observed were shown in the top-left corner of the composite images. (TIF) [file pgen.1005828.s008.tif]

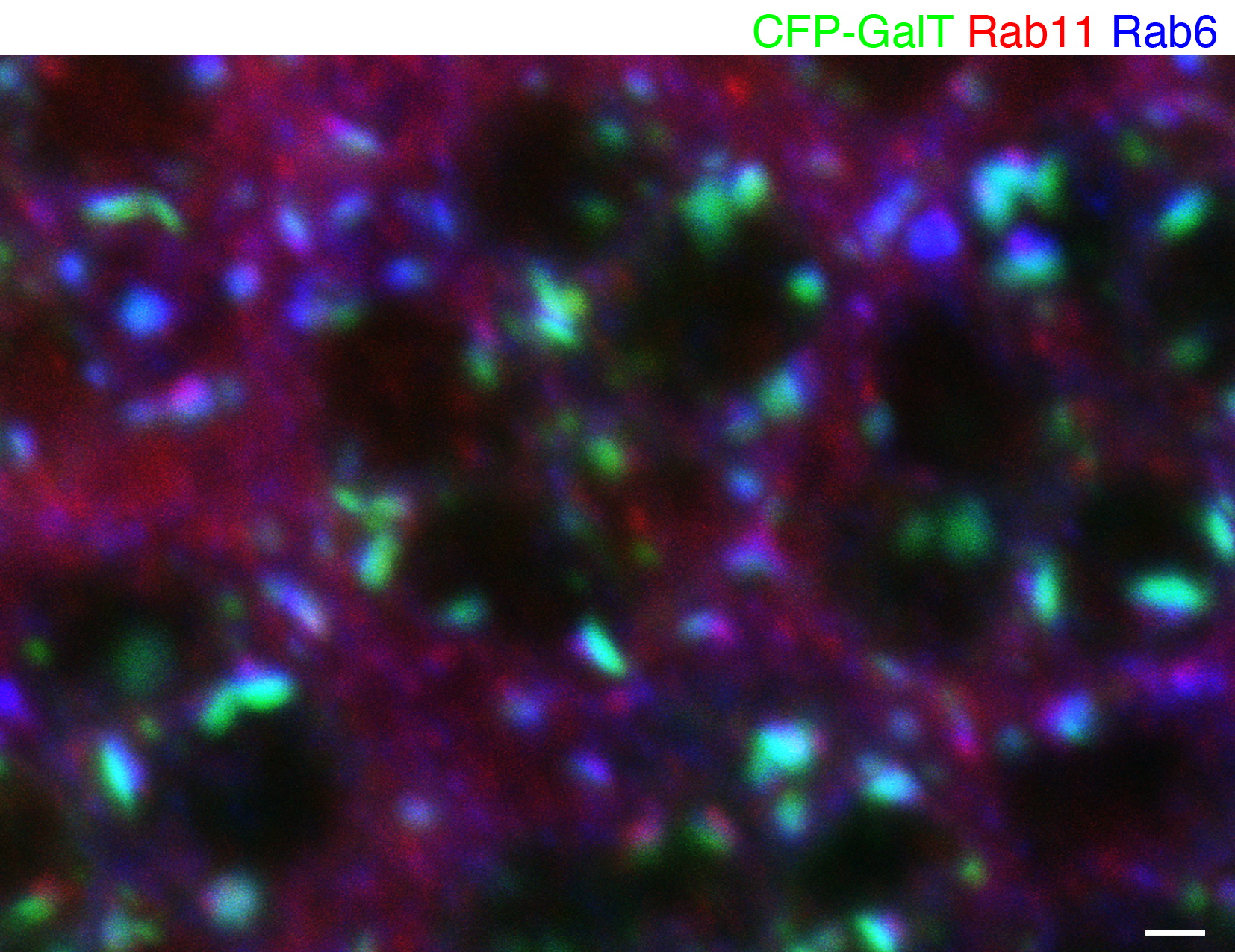

Supplement: S9 Fig — Immunostaining of a wild type eye expressing CFP-GalT (green) driven by GMR-Gal4 with anti-Rab11 (red) and anti-Rab6 (GP1) (blue). Scale bars: 1 μm. (TIF) [file pgen.1005828.s009.tif]
